# Two-photon nanoprobes based on bioorganic nanoarchitectonics with a photo-oxidation enhanced emission mechanism

**DOI:** 10.1038/s41467-023-40897-4

**Published:** 2023-08-26

**Authors:** Shukun Li, Rui Chang, Luyang Zhao, Ruirui Xing, Jan C. M. van Hest, Xuehai Yan

**Affiliations:** 1 State Key Laboratory of Biochemical Engineering, Institute of Process Engineering, Beijing, 100190 China; 2 Bio-Organic Chemistry, Institute for Complex Molecular Systems, Eindhoven University of Technology, P.O. Box 513 MB Eindhoven, The Netherlands; 3 School of Chemical Engineering, University of Chinese Academy of Sciences, Beijing, 100049 China; 4 Center for Mesoscience, Institute of Process Engineering, Chinese Academy of Sciences, Beijing, 100190 China

**Keywords:** Photobiology, Nanostructures, Nanostructures

## Abstract

Two-photon absorption (TPA) fluorescence imaging holds great promise in diagnostics and biomedicine owing to its unparalleled spatiotemporal resolution. However, the adaptability and applicability of currently available TPA probes, which act as a critical element for determining the imaging contrast effect, is severely challenged by limited photo-luminescence in vivo. This is particularly a result of uncontrollable aggregation that causes fluorescence quenching, and inevitable photo-oxidation in harsh physiological milieu, which normally leads to bleaching of the dye. Herein, we describe the remarkably enhanced TPA fluorescence imaging capacity of self-assembling near-infrared (NIR) cyanine dye-based nanoprobes (NPs), which can be explained by a photo-oxidation enhanced emission mechanism. Singlet oxygen generated during photo-oxidation enables chromophore dimerization to form TPA intermediates responsible for enhanced TPA fluorescence emission. The resulting NPs possess uniform size distribution, excellent stability, more favorable TPA cross-section and anti-bleaching ability than a popular TPA probe rhodamine B (RhB). These properties of cyanine dye-based TPA NPs promote their applications in visualizing blood circulation and tumoral accumulation in real-time, even to cellular imaging in vivo. The photo-oxidation enhanced emission mechanism observed in these near-infrared cyanine dye-based nanoaggregates opens an avenue for design and development of more advanced TPA fluorescence probes.

## Abstract (web summary)

Two-photon absorption (TPA) affords unparalleled spatiotemporal resolution for bioimaging, but the photo-oxidation tends to weaken the photoluminescence in vivo, limiting the usefulness of TPA probes. Here, the authors report self-assembling near-infrared cyanine dye-based nanoprobes of enhanced TPA fluorescence imaging capacity based on a photo-oxidation enhanced emission mechanism.

## Introduction

Two-photon absorption (TPA) fluorescence imaging has gained increasing prominence in the diagnostic field due to its intrinsically high spatiotemporal resolution, reduced out-of-focus photo-bleaching, diminished auto-fluorescence, and deeper tissue penetration^1–7^. Critical to the success of TPA fluorescence imaging is to apply TPA probes with robust luminescence and excellent biosafety, in order to be able to visualize biological processes at cellular levels, tissues and even organisms in real-time^8–11^. To meet these requirements, much effort has been put over the years in the optimization of the chemical structure of chromophores and the assembly of TPA probes in well-defined nanostructures. Both approaches have improved the photo-luminescence and biocompatibility to some extent^12–15^. Nevertheless, the clinical application of TPA probes is still limited because of the following reasons. First and foremost, sustainable luminescence of organic TPA molecular probes or nanoprobes (NPs) is compromised by oxidation during photo-irradiation, which diminishes their contrast effect^16^. Second, since most TPA molecules are based on extended conjugated compounds modified with donor and/or acceptor couples, they are prone to aggregation-caused quenching (ACQ) due to their propensity to stack, even when encapsulated in nanocarriers^17–21^. Luminogens with aggregation-induced emission (AIEgens), as the emerging chromophores for TPA fluorescence imaging, showed enhanced fluorescence upon aggregation owing to the restriction of intramolecular motions, which circumvented the ACQ effect^22–24^. Nonetheless, both types of chromophores require complex chemical modifications to increase their two-photon absorption crosssections (TPACSs). It should be noted that their biosafety remains a significant challenge. Not only these organic molecular probes require tedious synthetic routes, but also the potential toxicity (such as unclear metabolism) of inorganic NPs raises safe concerns for in vivo applications^25–29^. Hence, significant efforts should be undertaken to engineer biologically benign TPA probes with superior photoluminescence that remains unaffected by photo-oxidation and aggregation, particularly for in vivo TPA fluorescence imaging applications.

**Fig. 1:**
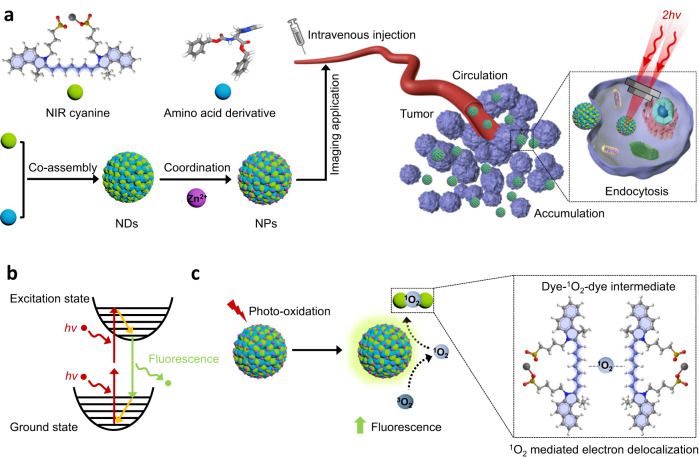
Schematic illustration of fabrication, applicability and mechanism of TPA NPs. **a** Amino acid derivatives and NIR cyanine dyes co-assembled into spherical NDs. The introduction of Zn^2+^ increased the structural stability to form NPs. TPA NPs are applicable for tumor imaging. Processes of NPs circulation, accumulation in tumor tissue and even single tumor cell endocytosis can be visualized. **b** Principle of two-photon excited fluorescence of chromophores. **c** Upon photo-oxidation, the NPs showed the enhanced TPA fluorescence emission, which was governed by the dye-^1^O_2_-dye intermediates. That is to say, ^1^O_2_-dimerized chromophore intermediates enhanced electron delocalization to simultaneously absorb two photons, and therefore enhanced the fluorescence emission.

Organic near-infrared (NIR) cyanine dyes, such as indocyanine green (ICG), possess high molar extinction coefficients. Therefore, they are applicable for clinical imaging applications^30,31^. Generally, NIR cyanine molecules contain two aromatic nitrogen-containing heterocyclic ring systems linked by a polymethine bridge. The positive charge on one nitrogen atom is involved in resonance delocalization with the second nitrogen, rendering extensive charge delocalization and thus giving a polar character to the entire molecule^32,33^, which may reflect its nonlinear optical behavior. Herein, we demonstrate that amino acid derivative-facilitated self-assembly of NIR cyanine dyes leads to a class of bioorganic TPA NPs capable of two-photon absorption enhancement. Intriguingly, singlet oxygen (^1^O_2_) generated in the process of photo-oxidation mediates cyanine chromophore dimerization, which enhances the electron delocalization and enlarges the nonlinear absorption of TPA NPs. The resulting TPA NPs possess unique photo-oxidation enhanced emission and considerable stability. More importantly, the TPACS and antiphoto-bleaching ability of cyanine-based TPA NPs are significantly improved when compared with the commonly used rhodamine B (RhB)^34^, therefore, high contrast imaging of visualizing blood circulation and tumoral accumulation of TPA NPs in real-time, even to cellular endocytosis in vivo can be achieved (Fig. 1). In comparison to the currently available TPA probes, our engineered NPs encompass the advantages of biosafety and unique photo-oxidation enhanced emission properties, which therefore provides a rationale for expanding the imaging modality of cyanine dyes and opens an avenue for design and development of more advanced TPA fluorescence probes.

## Results

### TPA NPs preparation and characterization

**Fig. 2:**
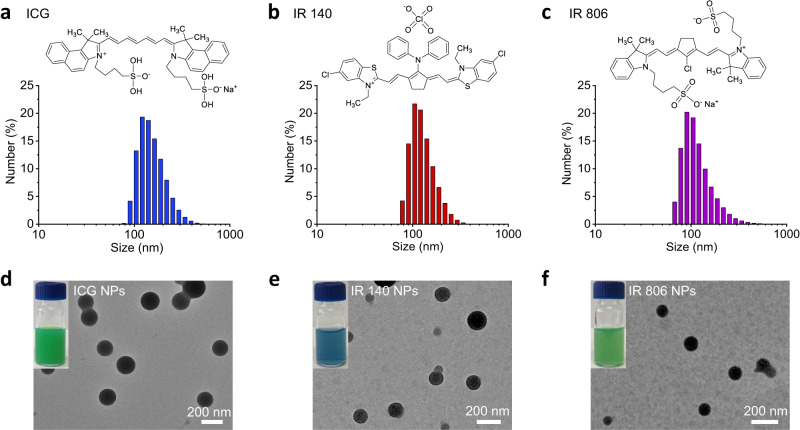
Morphology of TPA NPs created by co-assembly of histidine derivative ZHO and the different NIR dyes. DLS measurements and TEM images of **a**, **d** ICG NPs, **b**, **e** IR 140 NPs and **c**, **f** IR 806 NPs. The inserts indicate respective chemical structures of NIR cyanine dye molecules and optical pictures of TPA NPs. The concentration of ICG, IR 140 and IR 806 was kept the same at 125 µM.

Supramolecular self-assembly has been extensively used as a method to assemble NPs with novel optical properties^35,36^. In this regard, amino acid derivatives are versatile building blocks due to their ease of synthesis and wide variety of physicochemical features, and have thus attracted increasing attention for the design of these NPs^37^. In this study, the histidine (His) derivative Z-His-Obzl (ZHO) (Supplementary Fig. 1), was chosen as the template amino acid derivative to induce NIR cyanine dye ICG co-assembly due to its amphiphilic and positively charged nature. After mixing ICG aqueous solution with the ZHO dimethyl sulfoxide (DMSO) solution, an immediate turbid solution occurred. The stoichiometry of ICG and ZHO was selected as 0.125 mM and 2.020 mM, respectively, to achieve optimized encapsulation efficiency (EE, 96.9%) and loading efficiency (LE, 11.2%) of ICG molecules (Supplementary Tab. 1). However, transmission electron microscopy (TEM) image showed the turbid solution consisted of nanodroplets (NDs), termed as ICG NDs, that were kinetically-trapped unstable aggregates^38^ (Supplementary Fig. 2), further evidenced by the presence of crystalline precipitates after aging for 24 h (Supplementary Fig. 3). To stabilize the assembled nanostructures, zinc ions (Zn^2+^) were introduced to coordinate with the nitrogen atom of the imidazole group of ZHO to obtain more thermodynamically favorable ICG NPs, as evidenced by the Fourier transform infrared spectroscopy (FTIR) spectra result (Supplementary Fig. 4). Compared to the ZHO and ICG, the vibration of imidazole groups in ICG NPs shifted from 1693 cm^−1^ to 1718 cm^−1^, indicating the coordination of Zn^2+^ with imidazole^39^. As expected, the colloidal stability of the resulting ICG NPs was enhanced after aging for 24 h (Supplementary Fig. 3) and the concentration of Zn^2+^ was determined to be 0.438 mM. In addition, there was little difference between the ICG NDs and the ICG NPs in terms of spectral features, indicating a similar arrangement of the ICG molecules (Supplementary Fig. 5). The enhanced absorbance intensity of the ICG NPs further suggested that Zn^2+^ coordination improves structural robustness^40^ (Supplementary Fig. 5). The obtained ICG NPs possess average hydrated diameters of 159.8 ± 48.1 nm (Fig. 2a and Supplementary Tab. 2), as determined by dynamic light scattering (DLS). To demonstrate the universality, other NIR cyanine dyes, including IR 140 and IR 806 were co-assembled by the same method. The average sizes of IR 140 NPs and IR 806 NPs were found to be 132.2 ± 42.1 nm (Fig. 2b and Supplementary Tab. 2) and 123.5 ± 56.7 nm (Fig. 2c and Supplementary Tab. 2), respectively. TEM images further confirmed the spherical morphologies of ICG NPs, IR 140 NPs and IR 806 NPs (Fig. 2d–f), with sizes that were nearly identical to their DLS data.

**Fig. 3:**
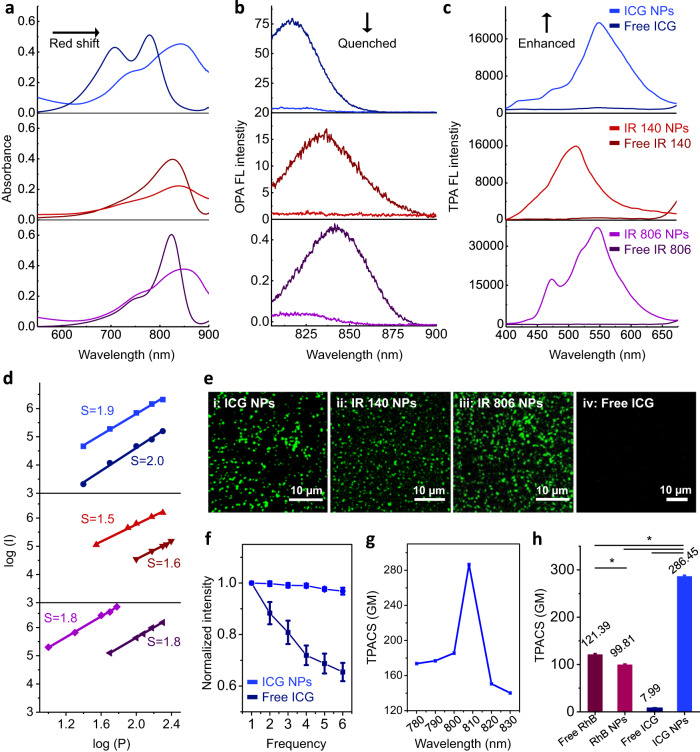
Optical properties of NIR cyanine dye-based NPs. **a** Absorption spectra, **b** OPA fluorescence (FL) spectra and **c** TPA FL spectra of free NIR cyanine dyes and their corresponding NPs. **d** Linear curve fitted by double exponential model between the emission intensity log (I) and excitation power log (P), where the “S” indicated the slope of fitted lines. **e** CLSM images of (i) ICG NPs, scale bar is 10 µm; (ii) IR 140 NPs, scale bar is 10 µm; (iii) IR 806 NPs, scale bar is 10 µm and (iv) free ICG, scale bar is 10 µm. All figures are obtained in the emission range of 495-540 nm. **f** The photo-stability test of free ICG and ICG NPs as a function of scanning frequency. The power density of laser excitation was 1.5 W cm^−2^. Error bars denote the standard deviation (*n* = 3 independent experiments). Data are presented as mean values +/− S.D. **g** TPACS of ICG NPs at different wavelengths using RhB dissolved in methanol as reference. Error bars denote the standard deviation (*n* = 3 independent experiments). Data are presented as mean values +/− S.D. **h** Comparison of TPACS of free RhB, RhB NPs, free ICG and ICG NPs. Error bars denote the standard deviation (*n* = 3 independent experiments). Data are presented as mean values +/− S.D., and *P* values are calculated by one-way ANOVA ^*^P < 0.05. The power density of laser excitation for all TPACS measurements was 0.1 W cm^−2^. The concentration of NIR cyanine dyes and RhB used in all figures was 25 µM.

Subsequently, the organization of the chromophores within the nanoparticles was investigated. Compared with their free state, NIR cyanine dye-based NPs showed a broadened and large red-shift in the absorption spectra (Fig. 3a), which can be attributed to the electron delocalization promoted by noncovalent interactions^41^. Of note, such intermolecular interactions significantly decreased their one-photon absorption (OPA) fluorescence intensity (810–900 nm), where the calculated fluorescence quenching efficiency of ICG NPs, IR 140 NPs and IR 806 NPs was 93.8%, 93.4% and 92.2%, respectively (Fig. 3b). As another main pathway of photo-activated NIR cyanine dyes^42^, photothermal relaxation of NPs was inferior than the corresponding free dyes as well (Supplementary Fig. 6). Remarkably, in response to femtosecond (fs) Ti: Sapphire oscillator 808 nm laser irradiation, NIR cyanine dye-based NPs exhibited drastically enhanced TPA fluorescence emission (400–650 nm) (Fig. 3c). Similar behavior was observed for all three investigated NIR cyanine molecules, but not for other dyes including porphyrins (protoporphyrin IX (PpIX), tetraphenylporphyrin tetrasulfonic acid (TPPS)) and phthalocyanines (nickel(II) phthalocyanine-tetrasulfonic acid tetrasodium salt (NiTSPc), naphthalocyanine (NaPc)) (Supplementary Fig. 7). All NPs were prepared with the same method and their TPA fluorescence was quenched when comparing with their free state. These comparative results suggested that the enhanced TPA fluorescence might be a unique property of NIR cyanine dyes. To understand the increased fluorescence upon TPA, this process was investigated in more detail. By changing the power energy, the TPA fluorescence spectra of free NIR cyanine dye-based NPs and the corresponding free dyes were recorded (Supplementary Fig. 8). The fluorescence of free NIR cyanine dyes displayed quadratic emission intensity as a function of increased incident power energy (Fig. 3d and supplementary Tab. 3), suggesting that the resonance structure of the cyanine dyes promoted the inherent push-pull electron transfer for TPA. Importantly, once NPs were formed, the TPA fluorescence intensity increased at least one order of magnitude when compared with free NIR cyanine dyes (Supplementary Fig. 8 and Fig. 3d), implying that the aggregated state enhanced the TPA fluorescence emission. Moreover, the TPA fluorescence emission was observed by employing a confocal laser scanning microscope (CLSM) equipped with a fs Ti: Sapphire oscillator laser at the wavelength of 808 nm and an emission channel of 495-540 nm, which covered the spectral emission ranging from 400 to 650 nm of the NIR cyanine dye-based NPs (Fig. 3e: i-iii). To demonstrate the importance of the aggregated state in the observation of the improved TPA and the broad applicability of this concept, we constructed a series of different NIR dye aggregates and compared them to the free state (Fig. 3e: iv). Besides, we obtained the dye complexes with proteins and polypeptides: ICG/bovine serum albumin (BSA) complex (Supplementary Figs. 9a and 10a), IR140/BSA NPs (Supplementary Figs. 9b and 10b) and ICG/poly(L-lysine) (PLL) NPs (Supplementary Figs. 9c and 10c). All assemblies showed enhanced TPA fluorescence, confirming that the aggregated state is a crucial feature to facilitate enhanced TPA fluorescence emission of cyanine dyes.

Due to the positive effect of nanostructures in keeping the stability of emitted fluorescence^43,44^, photo-bleaching damage was significantly alleviated. Taking ICG NPs as an example, superior anti-photo-bleaching stability, rather than rapid degradation as observed for free ICG, was observed (Fig. 3f). Also, the introduction of Zn^2+^ increased the TPA fluorescence stability (Supplementary Fig. 11). These results were mainly ascribed to structural protection associated with the formation of stable nanoarchitectonics. To gain a deeper insight into the highly unusual TPA optical properties of NIR cyanine dye-based nano-assemblies, comparative results of ICG with the organic fluorescent dye RhB (Supplementary Fig. 12a), commonly used as TPA fluorophore, were obtained. Prepared in the same way as the ICG NPs, RhB NPs possessed a uniform spherical morphology with an average diameter of 136.5 ± 51.3 nm (Supplementary Fig. 12b and c) and showed a broadened absorption peak with spectroscopy that was indicative of assembly formation (Supplementary Fig. 12d). Furthermore, a quenching effect in OPA fluorescence (Supplementary Fig. 12e) was observed. The TPA fluorescence spectra of RhB with variable excitation wavelength showed that the TPA fluorescence emission of RhB was located in the range of around 520–680 nm when simultaneously absorbing two photons and was independent of the excitation wavelength used. This observation indicated that a little bit energy loss was inevitable in the process of energy transition from excited state to ground state, thus leading to the deviation of the emitted photon from the theoretical energy with 2-fold frequency^1^ (Supplementary Fig. 12f). When subject to the excitation wavelength of 808 nm, it was found that the assembled RhB NPs showed a little red-shifted emission and slightly decreased intensity in comparison with free RhB (Supplementary Fig. 12g and h). This can be contributed to the ACQ effect of RhB, different from the observed phenomenon of enhanced TPA fluorescence in NIR cyanine dye. Further, the TPACS (δ), as the most commonly used parameter with a unit of Goeppert-Mayer (GM, 1 GM = 10^−50^ cm^4^·s photon^−1^) for characterizing TPA chromophores^45^, was quantitatively measured. ICG NPs showed a wavelength-dependent behavior and gave a maximum of 286.45 GM upon 808 nm laser excitation (Fig. 3g), which is 2.36-fold and 2.87-fold higher than free RhB and RhB NPs, respectively. Importantly, the TPACS of ICG NPs was 35.90-fold larger than free ICG (Fig. 3h and supplementary Tab. S4), indicating that ICG NPs are more useful in TPA fluorescence imaging in comparison to free ICG.

### Photo-oxidation enhanced emission mechanism of TPA NPs

**Fig. 4:**
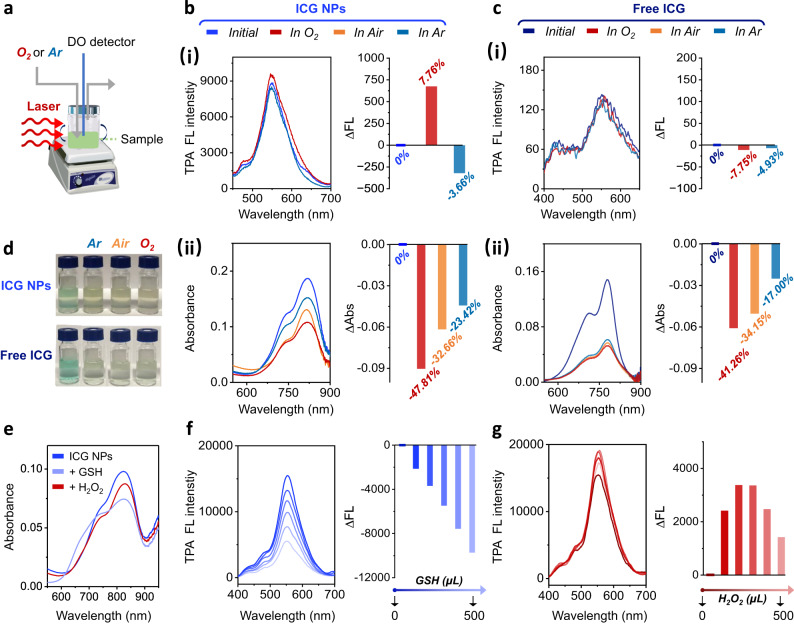
Photo-oxidation enhanced emission of ICG NPs. **a** Schematic illustration of ICG NPs in different oxygen-dependent atmospheres. **b** (i) TPA FL spectra and the (ii) absorption spectra of ICG NPs by laser irradiation (1.5 W cm^−2^, 10 min) in different oxygen-dependent atmospheres. **c** (i) TPA FL spectra and the (ii) absorption spectra of free ICG by laser irradiation (1.5 W cm^−2^, 10 min) in different oxygen-dependent atmospheres. The right in Fig. **b** (i) and **c** (i) indicated the ∆Fluorescence (∆FL) with the definition of FL−FL_initial_, and the value of ∆FL/FL_initial_ was noted. The right in Fig. **b** (ii) and **c** (ii) indicated the ∆Abs with the definition of Abs−Abs_initial_, and the value of Abs/Abs_initial_ was noted. **d** Optical pictures of ICG NPs and free ICG after laser irradiation (1.5 W cm^−2^, 10 min). **e** Absorption spectra of ICG NPs after adding reductant (GSH) and oxidant H_2_O_2_. TPA fluorescence spectra of ICG NPs as a function of **f** GSH and **g** H_2_O_2_. The concentration of ICG used in all figures was 25 µM.

Further studies were conducted to uncover the underlying mechanism governing the enhanced TPA fluorescence emission. Intriguingly, the fluorescence emission of ICG NPs and free ICG showed different behavior upon photo-oxidation (Fig. 4a). The fluorescence intensity upon laser irradiation of ICG NPs was enhanced, ∆FL/FL_initial_ = 7.67%, in aqueous solution saturated with O_2_ (dissolved oxygen concentration of 11.5 mg mL^−1^), while this change was opposite in aqueous solution saturated with Ar (dissolved oxygen concentration of 3.6 mg mL^−1^) with ∆FL/FL_initial_ = −3.66% (Fig. 4b-i). In contrast, only photo-degradation was observed when free ICG was irradiated under these different oxygen-containing conditions (In O_2_-rich condition: ∆FL/FL_initial_ = −7.75%; In Ar-rich condition: ∆FL/FL_initial_ = −4.93%) (Fig. 4c-i). In absorption spectroscopy, a color change of free ICG from green to yellow was observed and the loss in absorbance was proportional to the dissolved oxygen concentration: the ∆Abs/Abs_initial_ of free ICG in Ar-rich condition is −17.00% while its ∆Abs/Abs_initial_ in air condition and O_2_-rich condition is −34.15% and −41.26%, respectively (Fig. 4c-ii and d), confirming the photo-degradation phenomenon. A similar tendency in absorption spectra was observed for ICG NPs: the ∆Abs/Abs_initial_ of ICG NPs in Ar-rich condition is −23.42% while its ∆Abs/Abs_initial_ in air condition and O_2_-rich condition is −32.66% and −47.81%, respectively (Fig. 4b-ii and d), which thus differed from their fluorescence intensity enhancement. The inconsistency between fluorescence and absorbance motivated us to conjecture that the enhanced fluorescence emission is mediated by short-lived unstable intermediates. Alternatively, the TPA fluorescence can be easily controlled by other means, including addition of an O_2_ scavenger (glutathione, GSH) as well as elevating oxygen levels (addition of H_2_O_2_), that showed little influence on the assembled nanostructures (Fig. 4e). As displayed in Fig. 4f, the fluorescence emission of ICG NPs was diminished when GSH depleted dissolved oxygen or related active species, and their ∆FL/FL_initial_ was changed to −63.05% as the GSH volume increased up to 500 μL. Not surprisingly, the fluorescence emission showed oxygen-dependent photo-oxidation enhancement to some extent upon addition of H_2_O_2._ When the volume of H_2_O_2_ was below 300 μL, the ∆FL/FL_initial_ of ICG NPs changed as 24.55% while when the volume of H_2_O_2_ exceeded 300 μL to 500 μL, i.e., when excessive photo-oxidation was induced, chromophores in the ICG NPs degraded as well because their ∆FL/FL_initial_ decreased from 24.55% to 12.26% (Fig. 4g). These results again demonstrated the existence of oxygen-dependent unstable intermediates, which enhanced the TPA fluorescence.

**Fig. 5:**
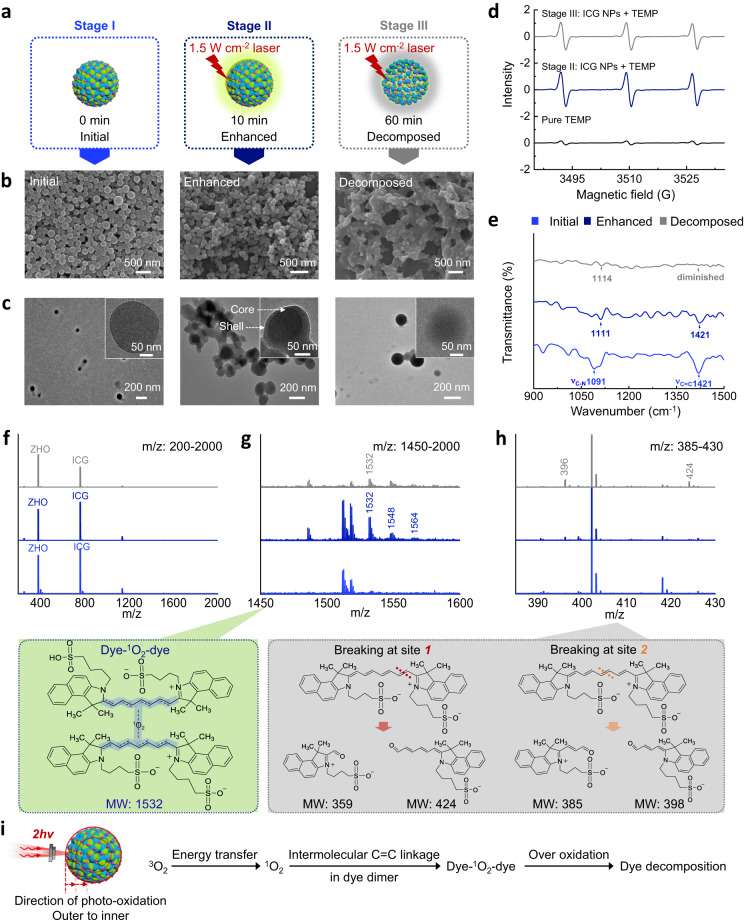
Proposed mechanism of photo-oxidation enhanced emission of ICG NPs. **a** Schematic illustration of initial, enhanced (1.5 W cm^−2^, 10 min) and decomposed (1.5 W cm^−2^, 60 min) stages of ICG NPs. Morphological characterizations of ICG NPs at different stages: **b** SEM images and **c** TEM images. **d** EPR spectra of ICG NPs at different stages. TEMP was added as indicating agent during laser irradiation. **e** FTIR transmittance spectra of ICG NPs at different stages. **f–h** ESI-MS of the ICG NPs and possible chemical structures at different stages. Fig. **f**, **g** and **h** denote the m/z range 200-2000, 1450-1600 and 385-430, respectively. **i** Schematic illustration of photo-oxidation enhanced emission mechanism. The concentration of ICG used in all figures was 25 µM.

In order to clarify the properties and structure of the intermediates, the fluorescence changes of ICG NPs were classified into initial, enhanced and decomposed stages according to the photo-oxidation process. The initial stage (termed as stage I) refers to the ICG NPs without laser irradiation. The enhanced stage (termed as stage II) refers to when ICG NPs were irradiated by laser (1.5 W cm^−2^, 10 min) to show the enhanced fluorescence. The decomposed stage (termed as stage III) means when no fluorescence of ICG NPs was detected after laser irradiation (1.5 W cm^−2^, 60 min) (Fig. 5a). In terms of morphology characterized by scanning electron microscope (SEM) (Fig. 5b) and TEM (Fig. 5c), spherical nanostructures with uniform size distribution were observed in stage I. Once photo-oxidation occurred and accompanied with enhanced TPA fluorescence emission, NPs seemed more adherent and crossed together. Additionally, in stage II, the structures evolved from spherical to core-shell. In stage III (excessive photo-oxidation), crossing between NPs further increased and core-shell structures disappeared. Based on the observed morphology changes in the photo-oxidation process, it can be inferred that degradation proceeded gradually from the outside to the inside of ICG NPs, and unstable intermediates responsible for enhanced TPA fluorescence emission may be generated as well. Obviously, oxygen is crucial in the photo-oxidation enhanced fluorescence emission and determines the structure of intermediates of ICG NPs. Therefore, the functional form of oxygen was detected by electron paramagnetic resonance (EPR). The ^1^O_2_ detector probe 2,2,6,6-tetramethyl-piperidin (TEMP), was used to react with ^1^O_2_ to form a stable nitroxide radical product 2,2,6,6-tetramethylpiperidine-1-oxyl (TEMPO), which can be recorded by EPR^46^. As shown in Fig. 5d, compared with the generation of TEMPO signal in the pure TEMP group, the intensity of TEMPO signal significantly increased in stage II. This suggested that the presence of ^1^O_2_ in ICG NPs promoted the formation of TEMPO. Importantly, the TEMPO signal intensity in stage II was higher than that in the stage III, implying that ^1^O_2_ indeed participated in the photo-oxidation mediated the enhancement of TPA fluorescence emission. Furthermore, FTIR spectra (Fig. 5e) and electrospray ionization mass spectra (ESI-MS) (Fig. 5f–h) of ICG NPs at different stages were recorded. The signal of the C = C stretching vibration of ICG molecules^47^ diminished from stage I to stage III, implying that C = C bonds were attacked by photo-oxidation. The bands corresponding to the C-N stretching vibration can be detected in three stages, suggesting that C-N bonds were not affected by photo-oxidation (Fig. 5e). In ESI-MS, a characteristic m/z of 1532 peak that was not observed in the stage I, appeared in stage II and was almost extinct in stage III (Fig. 5f and g). The m/z peaks, such as 398 and 424, which indicated fragment products by ^1^O_2_-mediated photo-degradation of ICG molecules at different C = C bonds^48^, were observed (Fig. 5h). Hence, the molecules corresponding to m/z = 1532 are intermediates that govern the enhanced fluorescence emission. Based on this analysis, a possible structure of these intermediates is listed in Fig. 5g, where two ICG molecules are linked by ^1^O_2_ and the linking sites are located at the C = C bond. This ^1^O_2_-containing intermediate contributed to photo-oxidation enhanced TPA fluorescence of ICG NPs, a unique characteristic of ICG NPs, quite different from the ICG dimer without connection by oxygen^49^. Molecular dynamics (MD) simulation results (Supplementary Fig. 13) supported our hypothesis: ZHO mediated self-assembly of ICG resulted in closer distance between neighboring ICGs. The ICG molecules exhibited a minimum intermolecular distance of approximately 3.3 Å, which facilitated electron delocalization for TPA fluorescence emission^50^ and paved the way for ^1^O_2_ to link their C = C bonds. Upon laser irradiation, ICG molecules that were excited to the T_1_ state transfer their energy to ^3^O_2_ by forming ^1^O_2_. Then, ^1^O_2_ intermolecularly conjugated the C = C bonds to form intermediates (dye-^1^O_2_-dye) within ICG NPs, further enlarging electron delocalization for TPA absorption and finally leading to enhanced fluorescence emission (Fig. 5i). This mechanism provides a plausible explanation for the observed enhancement of TPA fluorescence emission.

### Imaging application of TPA NPs

Given the fact that stability of NPs is important and has been a formidable challenge in biomedical imaging^51^, the stability of ICG NPs was investigated. In absorption spectroscopy (Supplementary Fig. 14), upon storage, the absorption peak of ICG NPs showed little alteration during 15 days of aging (normalized 16.44% decrease in intensity). By contrast, free ICG molecules underwent rapid degradation (normalized 54.51% decrease in intensity). Additionally, a red-shift appeared on the 15^th^ day, suggesting the free ICG was unstable and formed large aggregates^52^. These comparative results demonstrated that the multiple noncovalent interactions endowed the ICG NPs with colloidal stability. In addition, by creating 10-fold (v/v) dilutions of their suspensions with 10% fetal bovine serum (FBS) and incubating the suspensions at 37 °C for 24 h, the morphology of the ICG NPs showed no discernible change instead of a little size increase assigned to serum protein adsorption (Supplementary Fig. 15 and supplementary Tab. 5), suggesting excellent physiological stability. Considering the acidic microenvironment of tumors, stability test in mimicking tumoral acid-microenvironment was conducted as well. The absorption spectra of ICG NPs and RhB NPs were recorded in different pH conditions to monitor their stability (Supplementary Fig. 16). Apart from a slight absorption decrease, no band shift was observed in ICG NPs when decreasing pH value from 7 to 5. But a considerable turbidity decrease and a blue-shift were observed in RhB NPs, indicating their disassembly^53^. The disassembly propensity of RhB NPs can be attributed to pH-susceptibility of RhB-based dyes^54^. By contrast, the less susceptibility of ICG NPs to pH variation highlighted their advantages in tumor imaging application.

**Fig. 6:**
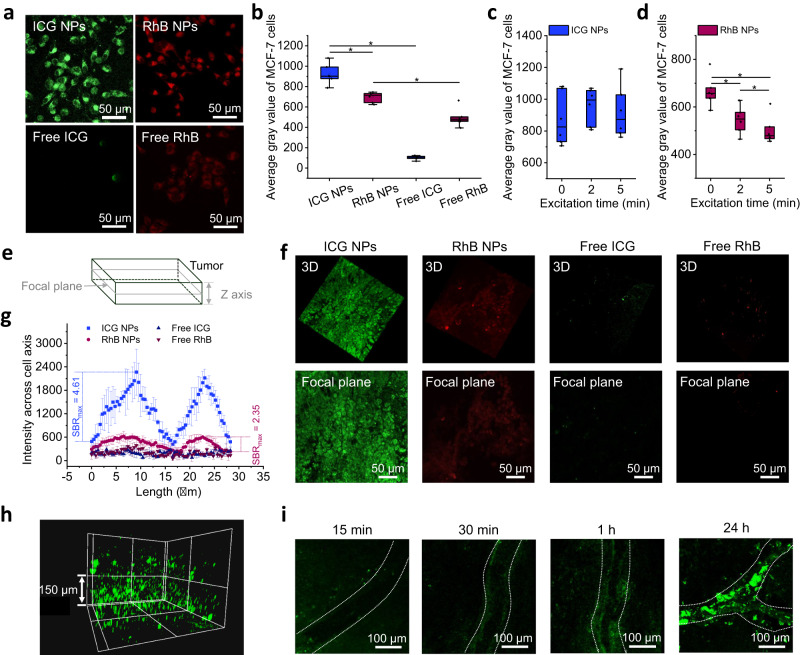
In vitro and in vivo applicability of nanoprobes. **a** Representative CLSM images and **b** calculated average gray values of MCF-7 cells that incubated with four kinds of formulations including ICG NPs, free ICG, RhB NPs and free RhB (dye concentration: 25 µM) for 24 h. Error bars denote the deviation (*n* = 6 biologically independent cells). Data are presented as mean values +/− S.D., and *P* values are calculated by one-way ANOVA ^*^P < 0.05. Calculated average gray value of MCF-7 cells in **c** ICG NPs and **d** RhB NPs over excitation time (0, 2, 5 min). The power density of laser excitation was 1.5 W cm^−2^. Error bars denote the deviation (*n* = 6 biologically independent cells). Data are presented as mean values +/− S.D., and *P* values are calculated by one-way ANOVA ^*^P < 0.05. **e** Schematic illustration of tumor tissue imaging in vivo. **f** Representative three-dimensional reconstruction images of tumor section along *Z* axis (upper) and focal plane images (lower), where the Z depth is 20 µm. Mice bearing MCF-7 tumor were intravenously injected with ICG NPs, free ICG, RhB NPs and free RhB (dye dosage: 250 µM, 200 µL) for 24 h. The scale bar is 50 µm. **g** Fluorescence intensity cross cell axis of MCF-7 cells obtained from Fig. **f**. Error bars denote the deviation (*n* = 20 biologically independent cells). Data are presented as mean values +/− S.D., and *P* values are calculated by one-way ANOVA ^*^P < 0.05. **h** Three-dimensional reconstruction of tumor tissue after intravenous injection of ICG NPs for 24 h. The scale bar is 150 µm. **i** Representative CLSM images of real-time readout process of ICG NPs accumulation in blood vessels within tumor sites at different time intervals (15 min, 30 min, 1 h and 24 h). The white line indicates the blood vessel. The scale bar is 100 µm.

Next, the in vitro and in vivo application potentials of ICG NPs were assessed by comparing to free ICG, RhB NPs and free RhB. A standard 3-(4,5-dimethylthiazolyl-2)-2,5-diphenyltetrazolium bromide (MTT) cell survival assay regarding cytotoxicity of four different formulations was conducted (Supplementary Fig. 17). Cell viability was not affected when incubating the cells with NPs at the concentration range from 0 µM to 200 µM. Moreover, no significant difference of cell viability was observed between NPs group and their free state group. These results indicated their appreciable biocompatibility, paving the way for their biological application. After incubating cells with free dyes and NPs for 24 h, CLSM images (Fig. 6a and b) demonstrated that the dyes were most likely endocytosed in the cytoplasm, and the NPs (ICG NPs and RhB NPs) showed the much higher fluorescence intensity than free dyes (free ICG and free RhB), suggesting that nanostructures increased the endocytosis efficiency of dyes. By comparison, the fluorescence intensity of RhB NPs in the cytoplasm was inferior to ICG NPs, which was attributed to their lower δ value and reduced stability in acidic conditions. Furthermore, the photo-bleaching test regarding ICG NPs and RhB NPs was conducted as well. Human breast cancer MCF-7 cells were continuously excited and their average gray was recorded at 0 min, 2 min and 5 min to indicate the fluorescence intensity. During continuous photo-bleaching, ICG NPs in the cellular cytoplasm maintained robust photo-luminescence even after being irradiated up to 5 min (Fig. 6c), which can be mainly attributed to their photo-oxidation enhanced fluorescence emission and physiological stability. In contrast, RhB NPs showed distinct behaviors as they were gradually bleached over excitation time (Fig. 6d). As the in vitro experiments showed robust TPA fluorescence emission and superior anti-photo-bleaching capbility, ICG NPs served as excellent candidates for the imaging implementation in vivo.

The in vivo imaging was performed in tumor-bearing mice. When the tumor volume reached 150 mm^3^, NPs (ICG NPs, RhB NPs) and their free state formulations (free ICG and free RhB) with the same dye dosage (250 µM, 200 µL) were intravenously injected into mice. At 24 h post-injection, the mouse was anesthetized and the tumor site was imaged. From the focal plane, lower and upper sections were scanned along the *Z* axis (Fig. 6e). As shown in Fig. 6f, the fluorescence signal obtained at the cellular level can be obtained, where the upper panel showed the three-dimensional reconstruction images of tumor section with Z depth of 20 µm, and the lower plane showed the focal plane images. Cell entities can be identified in groups of ICG NPs and RhB NPs, but not in free ICG and free RhB groups, suggesting that NPs accumulated in tumor sites while free dyes may be quickly cleared from mice body^55^. Importantly, the imaging of ICG NPs within tumor cells was clearer than that of RhB NPs, verifying the intense fluorescence emission in vivo of ICG NPs. Also, the TPA fluorescence imaging of ICG NPs has been demonstrated in other three kinds of mice xenograft tumor models including mouse breast carcinoma cell line 4T1, mouse colorectal carcinoma cell line CT26 and human cervical cancer cell line Hela (Supplementary Fig. 18), indicating pervasive application of ICG NPs to other types of tumors. Moreover, the maximum signal-to-background ratio (SBR_max_) cross cell axis, to represent imaging contrast, was analyzed. The SBR_max_ of ICG NPs was calculated as 4.61, which is 1.96-fold higher than the counterpart value of RhB NPs (SBR_max_ = 2.35) (Fig. 6g). Besides, the three-dimensional scanning enabled the visualization of the distribution of ICG NPs in tumor tissue (Fig. 6h and supplementary Mov. 1), where the length along the *Z*-axis reached around 300 µm. Intriguingly, a real-time readout of the presence of ICG NPs within tumor vascular circulation was realized (Fig. 6i). Blood vessels in tumor tissue showed a relatively dark state about 15 min after intravenous injection. Over time, however, ICG NPs circulated to the tumor vascular sites and accumulated at the tumor vascular walls, which indicated an enhanced permeability and retention (EPR) effect. Further, ex vivo imaging of tumor biopsies (Supplementary Fig. 19), demonstrated that ICG NPs were endocytosed by cells without damaging their morphology, predicting that ICG NPs may be safe. Finally, OPA fluorescence imaging was conducted to compare the property of TPA fluorescence. Information regarding the distribution of NPs in different intact tissues at 24 h postinjection can be collected, which also confirmed that the ICG NPs possessed EPR effect within tumor (Supplementary Fig. 20). More detailed information, such as the cellular distribution within tumor tissue, cannot be provided by OPA fluorescence imaging technique. The comparisons emphasized the advantages of ICG NPs-based TPA fluorescence imaging: (i) The chromophores only at the focus point can be excited in TPA fluorescence imaging, which is the inherent nature of TPA imaging technique and guaranteed high imaging resolution; (ii) The high contrast imaging at the cellular level in vivo can be obtained owing to their high TPA cross-section, excellent stability, considerable anti-photo-bleaching capability and outstanding SBR of ICG NPs.

## Discussion

We have demonstrated that NIR cyanine dye-based nanoarchitectonics formed by amino acid derivative-facilitated self-assembly represents a versatile class of bioorganic TPA NPs with a photo-oxidation enhanced emission mechanism, which has not been previously reported. Singlet oxygen generated during photo-oxidation mediated chromophore dimerization, enhanced electron delocalization and enlarged nonlinear absorption of the TPA NPs, which are responsible for the enhanced TPA fluorescence emission. The resulting TPA NPs showed considerable colloidal stability and outstanding in vivo stability and compatibility, and more importantly, their TPA cross-section and anti-bleaching ability are more favorable than the benchmark dye RhB. These NPs show outstanding imaging performance both in vitro and in vivo.

Compared to the existing TPA probes, which are challenged by potential biotoxicity and limited photo-luminescence resulting from uncontrollable aggregation and inevitable photo-oxidation in harsh physiological milieu, the advantages of NIR cyanine dyes-based NPs are multifaceted (Supplementary Tab. 6): (i) The NPs successfully circumvent the impediments of ACQ and photo-degradation during biological imaging. That is, different from the chemically synthesized probes that were challenged by ACQ effect, the cyanine dye NPs showed enhanced TPA fluorescence in an aggregated state. Further, the photo-oxidation enhanced TPA fluorescence mechanism enabled NPs to emit robust fluorescence during photo-irradiation, thereby achieving high imaging contrast. (ii) The NPs, started from the amino acid derivative and broadly used cyanine dyes, have been demonstrated to possess outstanding biosafety, while the widely studied TPA probes (such as polymers and AIEgens) to some extent suffered from potential toxicity presumably due to the complex chemical modification to the chromophores. (iii) The employed fabrication strategy, avoiding the tedious chemical synthesis, is simple and versatile. Taken together, the resulting NPs not only repurposed cyanine dyes as TPA imaging NPs but also hold much potential in the field of clinical diagnosis.

## Methods

### Materials

ZHO was purchased from Bachem UK Ltd. ICG, IR 806, IR 140, RhB, ZnCl_2_, methanol were purchased from Sigma-Aldrich Inc. BSA was purchased from Solarbio Biotechnology Co. Ltd. MCF-7 cells (catalog number SCSP-669S), 4T1 cells (catalog number SCSP-5056), CT26 cells (catalog number TCM37) and Hela cells (catalog number SCSP-504) were provided by the National Collection of Authenticated Cell Cultures. Dulbecco’s Modified Eagle’s Medium (DMEM), Roswell Park Memorial Institute 1640 (RPMI 1640), heat-inactivated fetal bovine serum (FBS), Dulbecco’s phosphate-buffered saline (PBS), trypsin-EDTA, and penicillin-streptomycin were purchased from BioLegend Co. Other materials were purchased from Beijing Chemical Co. Ltd. unless otherwise noted.

### Preparation of NPs

ICG NPs were typically prepared as follows: 10 µL DMSO solution of ZHO (264 mM) was mixed with 985 µL aqueous solution of ICG (0.131 mM), followed by the addition of 5 µL ZnCl_2_ solution (100 mM) solution into the above mixed solution. The concentration of ZHO, ICG and Zn^2+^ was 2.640 mM, 0.129 mM and 0.500 mM, respectively. Based on the opalescence of the samples, nanostructures formed immediately and the pH value was 7, approximately. The obtained ICG NPs were stored at 4 °C in the dark for 24 h. The aged NPs were centrifuged with a centrifugal force (RCF) of 9391 g for 10 min and were dispersed in pure water.

RhB NPs, IR 806 NPs, TPPS NPs and NiTSPc NPs were prepared with the same above method. The concentration of all dyes in the final prepared NPs was kept same.

IR 140 NPs were prepared as follows: 10 µL DMSO solution of ZHO (264 mM) was mixed with 10 µL DMSO solution of IR 140 (13.100 mM), then 975 µL pure water and 5 µL Zn^2+^ (100 mM) solution were added to the stirred solution to obtain IR 140 NPs with a pH value around 7. The concentration of ZHO, IR 140 and Zn^2+^ was 2.640 mM, 0.129 mM and 0.500 mM, respectively. The aging and washing procedures of IR 140 NPs were same to ICG NPs.

PpIX NPs were prepared following the protocol same to IR 140 NPs. The concentration of PpIX in the formation of NPs was kept same to that of IR 140 NPs.

NaPc NPs were prepared as follows: 10 µL tetrahydrofuran (THF) solution of ZHO (264 mM) was mixed with 10 µL THF solution of NaPc (13.100 mM), then 975 µL pure water and 5 µL Zn^2+^ (100 mM) solution were added to the stirred solution to obtain NaPc NPs with a pH value around 7. The aging and washing procedures of NaPc NPs were same to ICG NPs.

### Morphological and spectral characterization

An aliquot of a suspension of TPA NPs was spread on a silica plate and totally dried in vacuum at room temperature. S-4800 (Hitachi, Japan) with 10 kV accelerating voltage was used for SEM measurements. TEM was performed by a JEM-1011 (JEOL, Japan) at 100 kV with a drop of sample carefully applied to a carbon-coated copper grid and dried in vacuum. The size distribution and zeta potential were determined using a Zetasizer Nano (Malvern, England). CLSM images were acquired by an FV500 confocal laser scanning microscope (Olympus, Japan) equipped with a Ti: Sapphire oscillator laser (Mai Tai, USA). TPA NPs were excited by the adjustable Ti: Sapphire oscillator laser and the signal channels used were 495–540 nm and 575–630 nm. The absorption spectra were recorded using a UV-2600 spectrophotometer (Shimadzu, Japan) with a quartz cuvette of 1 mm path length. The F-4500 fluorescence spectrometer (Hitachi, Japan) equipped with Xenon lamp as excitation source was used to measure the OPA fluorescence spectra of the samples with a quartz cuvette of 1.0 cm. The TPA fluorescence spectra were measured on a home-made optical platform. For excitation, an adjustable fs Ti: Sapphire oscillator laser (100 fs, SP-5W, Spectra physics, America) equipped with a short-pass filter (730 nm) was applied onto the sample in a 1.0 cm quartz cuvette, and spectra were recorded by an Omni-λ300 monochromator/spectrograph (Zolix, China) equipped with a PMTH-S1C1-CR131 photomultiplier tube. EPR measurements were conducted on the ESP-300 spectrometer (Bruker, America), which equipped with 808 nm laser at room temperature. TEMP agent was added into the samples to capture the singlet oxygen signal. FTIR spectra were recorded by the TENSOR 27 FTIR spectrometer (Bruker, America), with the samples prepared using the KBr pellet method. The molecular weight mass charge ratio (m/z) of ICG NPs were determined by the SolariX ESI-MS (Bruker, America). For photothermal relaxation test, the temperature increase was recorded every 60 s with the UT320 digital thermometer (UNI-T, China), and samples were irradiated by 808 nm laser with a power density of 1.5 W cm^−2^.

### Quantitative component analysis of ICG NPs

To determine the concentration of ICG and ZHO, the suspension solution of ICG NPs was centrifuged at a RCF of 9391 g for 20 min, and the precipitate was suspended in methanol. The absorption intensity of ICG was measured by absorption spectroscopy and the corresponded concentration of ICG molecules was determined by calibration absorption curves with a range of known standard concentrations. ZHO concentration were analyzed by Ultimate3000 high-pressure liquid chromatography (HPLC, Thermo Fisher Scientific, America) using calibration absorption curves with a range of known standard ZHO concentrations. To determine the concentration of Zn^2+^, the suspension solution of ICG NPs was centrifuged at a RCF of 9391 g for 20 min, and the precipitate was re-suspended by pure water and centrifuged again to remove free Zn^2+^ not participated in the assembly. The obtained precipitates were dissolved in 1% nitric acid solution. Then, the concentration of Zn^2+^ was measured by inductively coupled plasma-optical emission spectroscopy (ICP-OES, Leeman, America) and the corresponded concentration of Zn^2+^ was determined by calibration absorption curves with a range of known standard concentrations. The drug encapsulation efficiency (EE) and loading efficiency (LE) were calculated according to the following formula (1) and (2), respectively:

1 $${{\mbox{EE}}}=\frac{{{{\mbox{weight}}}} \,{{{\mbox{of}}}}\,{{{\mbox{ICG}}}} \,{{{\mbox{in}}}} \,{{{\mbox{the}}}} \,{{{\mbox{precipitate}}}}}{{{{\mbox{weight}}}}\, {{{\mbox{of}}}} \,{{{\mbox{ICG}}}} \,{{{\mbox{added}}}}}\times 100\%$$

2 $${{\mbox{LE}}}=\frac{{{{\mbox{weight}}}} \,{{{\mbox{of}}}} \,{{{\mbox{ICG}}}}\, {{{\mbox{in}}}} \,{{{\mbox{the}}}} \,{{{\mbox{precipitate}}}}}{{{{\mbox{weight}}}} \,{{{\mbox{of}}}} \,{{{\mbox{the}}}} \,{{{\mbox{precipitate}}}}}\times 100\%$$

### TPACS measurement

TPACS were measured with RhB in methanol as a reference^56^. The fs Ti: Sapphire oscillator laser was used. The TPA fluorescence spectra were recorded in a 1.0 cm quartz cuvette, where the ICG NPs concentration was kept as 20 μM in methanol (sample) and RhB NPs concentration was kept as 2 μM in methanol. The experimental fluorescence excitation and detection wavelengths (400 nm–700 nm) of samples and references were kept constant. The TPACS (δ) of the probes was calculated at each wavelength according to the following formula (3):

3 $${\delta }_{{{{{{\rm{sample}}}}}}}={\delta }_{{{{{{\rm{reference}}}}}}}\frac{{{\varnothing} }_{({{{{{\rm{reference}}}}}})}{I}_{({{{{{\rm{sample}}}}}})}{C}_{({{{{{\rm{reference}}}}}})}{\eta }_{({{{{{\rm{sample}}}}}})}^{2}{P}_{({{{{{\rm{reference}}}}}})}^{2}}{{\varnothing }_{({{{{{\rm{sample}}}}}})}{I}_{({{{{{\rm{reference}}}}}})}{C}_{({{{{{\rm{sample}}}}}})}{\eta }_{({{{{{\rm{reference}}}}}})}^{2}{P}_{({{{{{\rm{sample}}}}}})}^{2}}$$

Where *I* is the integrated fluorescence intensity, *C* is the concentration, *η* is the refractive index, *∅* is the quantum yield, and *P* is the incident power on the sample, subscript “reference” indicates reference and “sample” indicates sample, respectively.

### Mechanism demonstration of photo-oxidation enhanced emission

A suspension of ICG NPs with a concentration of 25 µM was added to a 1.0 cm quartz cuvette with a sealed cap. The solution was saturated with high-purified argon (Ar) or oxygen (O_2_) gas. The JPBJ-608 portable apparatus (INASE Scientific Instrument, China) was used to detect the dissolved oxygen concentration. ICG NPs solution in air atmosphere was used as control. Alternatively, GSH (5 mM), H_2_O_2_ and H_2_O were added to the ICG NPs solution to mimic the oxygen-depleted, oxygen-saturated, and control groups, respectively.

### Computational simulation

MD simulation and energy analysis were performed using *Gromacs (Version 5.1.4)* package^57^. The force field of small molecles including ICG and ZHO were constructed by *antechamber* program in *Ambertools16* package^58^ and *acpype.py* program^59^. The atomic charges of these molecules were fitted by DFT calculation under the restrained electrostatic potential (RESP) formalism. Water was modeled using the tip3p potential. The binary system for MD simulation consisted of 12 ICG and 120 ZHO that were randomly distributed in a water box sized 11 × 11 × 11 nm^3^, which was charge-neutralized by Na^+^ ions. The system was firstly minimized utilizing the conjugate-gradient algorithm, and then equilibrated through running for 100 ps NVT and NPT simulations sequentially. Production runs in the NPT ensemble at 298 K were run 60 ns employing the leapfrog algorithm with a time step of 2 fs to integrate the equations of motion. The electrostatic forces were treated with the particle-mesh Ewald (PME) approach. Both the cutoff values of van der Waals forces and electrostatic forces were set to be 1.2 nm. The LINCS algorithm was utilized to preserve bonds.

### Stability and biocompatibility

The physiological stability test was conducted by incubating ICG NPs at 37 °C for 24 h in 10-fold (v/v) dilutions of ICG NPs suspensions with FBS. Morphological changes were characterized by TEM and DLS. The anti-acidic stability of NPs was conducted as follows. The NPs were respectively put in pH 5 and pH 7 solutions, then their absorption spectra were recorded.

Biocompatibility of NPs was verified by a standard MTT cell survival assay. MCF-7 cells were seeded in 96-well plates (1 × 10^4^ cells well^−1^) and incubated for 24 h. Then, the media were replaced with 200 μL of DMEM containing different concentrations of ICG NPs, RhB NPs, free ICG and free RhB. After incubation for another 24 h, the cells were washed three times with PBS, infused with fresh media and the cell viability was examined.

### In vitro TPA fluorescence imaging

In vitro TPA fluorescence imaging was conducted as follows. MCF-7 cells were cultured in DMEM containing 10% FBS. They were seeded onto Petri dishes (5 × 10^3^ cells well^−1^) and incubated for 24 h at 37 °C in humidified ambiance of 5% CO_2_. Then, the medium was replaced with 2 mL of DMEM medium containing ICG NPs, free ICG, RhB NPs and free RhB (dye concentration: 25 μM). Then MCF-7 cells were incubated for 24 h at 37 °C. The intracellular localization of ICG was determined using an Olympus two-photon confocal laser scanning microscope (FVMPE-RS, Japan). All NPs (ICG NPs and RhB NPs) and their corresponding free states (free ICG and free RhB) were excited at 808 nm. The signal channel of ICG NPs and free ICG was located in 495–540 nm, while the signal channel of RhB NPs and free RhB was located in 575–645 nm.

### In vivo TPA fluorescence imaging

Animal procedures were approved by the Ethics Committee of the Institute of process engineering, Chinese Academy of Sciences (permit number: IPEAECA2018061). Female BALB/c-nude mice (6-8 weeks old, Beijing HFK Bioscience Co. Ltd., China) were housed in an environmentally controlled animal facility (temperature 23 °C, humidity 55 ± 5%) with regular 12/12 cycle. MCF-7 cells were collected and suspended in PBS with a concentration of 6 × 10^7^ cells mL^−1^. Each mouse was injected with 100 µL cellular suspension in the right sub-dermal dorsal area. The tumor dimensions were measured using a caliper every day. The tumor volume was determined using the following formula (4):

4 $${{{{{\rm{Tumor}}}}}}\,{{{{{\rm{volume}}}}}}=\frac{{{{{{\rm{length}}}}}}\times {{{{{\rm{width}}}}}}\times {{{{{\rm{width}}}}}}}{2}$$

Approximately 1 week after inoculation, the tumors approximately grew to the volume of 150 ± 30 mm^3^, the mice xenograft MCF-7 tumor models were established. Other three kinds of mice xenograft tumor models including 4T1 tumor, CT26 tumor and Hela tumor were established with the same above method.

For in vivo TPA imaging, 200 μL 5% glucose solution of ICG NPs, free ICG, RhB NPs and free RhB (dye concentration: 250 µM) were intravenously injected into the tumor bearing mice via the tail vein. After injection, the mice were anesthetized with 4% (w/w) chloral hydrate (10 mL kg^−1^ body) and the skin of tumor was peeled off. All fluorescence of NPs (ICG NPs and RhB NPs) and their corresponding free states (free ICG and free RhB) was determined using the Olympus two-photon confocal laser scanning microscope and excited at 808 nm. The signal channel of ICG NPs and free ICG was located in 495–540 nm, while the signal channel of RhB NPs and free RhB was located in 575–645 nm. For cellular imaging in vivo, an area of 300 × 300 µm was randomly selected as site of interest to perform the *Z* axis analysis and focal plane analysis. For imaging depth measurement to determine the spatial distribution of ICG NPs, the *Z* axis crosscutting was continued until the signal disappeared. For flow and accumulation of ICG NPs in tumor tissue, a blood vessel included-area around 430 × 430 µm was selected as site of interest to image at different time intervals. All obtained images were analyzed by the equipped software. For OPA fluorescence imaging, mice were anesthetized and scanned by an in vivo IVIS spectrum imaging system (PerkinElmer, America) at 24 h post-injection of ICG NPs. The signal of ICG was collected.

### Histological analysis

Tumor tissues were excised from the mice after imaging of ICG NPs accumulation after 24 h. The frozen tissue was sliced into 20 µm slices which were analyzed by Olympus two-photon confocal laser scanning microscope.

### Statistical Analysis

Statistical significance was determined using one-way analysis of variance (ANOVA) method. The data are presented as mean values +/− S.D., and *P* values are calculated by one-way ANOVA ^*^P < 0.05.

### Statistics and Reproducibility

Each experiment was repeated 3 times independently with similar results.

### Reporting summary

Further information on research design is available in the Nature Portfolio Reporting Summary linked to this article.

### Supplementary information

Supplementary Information

Reporting Summary

Description of Additional Supplementary Files

Supplementary Movie 1

## Data Availability

The Source data for main and supplementary figures generated in this study have been deposited in Figshare (https://figshare.com/s/15318812234a7e3721ba). The full image dataset is available from the corresponding author upon request. The remaining data are available within the Article, Supplementary Information or Source data file. Source data are provided with this paper.
