# Supplementary material for: Two-photon nanoprobes based on bioorganic nanoarchitectonics with a photo-oxidation enhanced emission mechanism
Source: Nat Commun. 2023 Aug 26;14:5227. doi: 10.1038/s41467-023-40897-4 (PMC10460436; doi:10.1038/s41467-023-40897-4)
Supplement: Supplementary file 1 — Supplementary Information [file 41467_2023_40897_MOESM1_ESM.pdf]

Supplementary Information

Two-Photon Nanoprobes Based on Bioorganic Nanoarchitectonics with a Photo-oxidation Enhanced Emission Mechanism

Shukun Li[1, 2], Rui Chang[1], Luyang Zhao[1], Ruirui Xing[1,3*], Jan C. M. van Hest[2], Xuehai Yan[1,3,4*]

[1] State Key Laboratory of Biochemical Engineering, Institute of Process Engineering, Beijing 100190, China

[2] Bio-Organic Chemistry, Institute for Complex Molecular Systems, Eindhoven University of Technology, P.O. Box 513, MB Eindhoven, The Netherlands

[3] School of Chemical Engineering, University of Chinese Academy of Sciences, Beijing 100049, China

[4] Center for Mesoscience, Institute of Process Engineering, Chinese Academy of Sciences, Beijing 100190, China

Captions for Supplementary Figures

Supplementary Fig. 1

Chemical structure of ZHO.

Supplementary Fig. 2

TEM image of ICG NDs.

Supplementary Fig. 3

Picture of ICG NDs and ICG NPs before and after aging for 24 h. The concentration of ICG was 125 μM.

Supplementary Fig. 4

FTIR transmittance spectra of ZHO, ICG and ICG NPs.

Supplementary Fig. 5

Absorption spectra of ICG NDs and ICG NPs after aging for 24 h. The concentration of ICG was 125 μM.

Supplementary Fig. 6

Photothermal relaxation of **a** free ICG and ICG NPs, **b** free IR 140 and IR 140 NPs and **c** free IR 806 and IR 806 NPs. The concentration of NIR cyanine dyes was 25 μM. Samples were excited by 808 nm laser with a power density of 1.5 W cm^{-2}.

Supplementary Fig. 7

TPA fluorescence spectra of free porphyrin-based dyes (**a** PpIX; **b** TPPS), phthalocyanine-based dyes (**c** NiTSPc; **d** NaPc) and their corresponding NPs, where the PpIX was excited at 780 nm, TPPS was excited at 780 nm, NiTSPc was excited at 780 nm and NaPc was excited at 800 nm. The insert in spectra showed the prepared NPs. The concentration of all dyes used in all figures was 25 μM.

Supplementary Fig. 8

TPA fluorescence spectra of free NIR cyanine dyes and their corresponding NPs as a function of excitation power. The concentration of NIR cyanine dyes and RhB used in all figures was 25 μM.

Supplementary Fig. 9

TEM images of **a** ICG/BSA complex, **b** IR 140/BSA NPs and **c** ICG/PLL NPs.

Supplementary Fig. 10

CLSM images of **a** ICG/BSA complex, scale bar is 50 μm, **b** IR 140/BSA NPs, scale bar is 10 μm, and **c** ICG/PLL NPs, scale bar is 10 μm. All figures are obtained in the emission range of 495-540 nm.

Supplementary Fig. 11

TPA fluorescence spectra of ICG NDs and ICG NPs after aging for 24 h. The concentration of ICG was 25 μM.

Supplementary Fig. 12

a Chemical structure of RhB. **b** DLS measurements of RhB NPs. **c** TEM image of RhB NPs. The insert shows a picture of the RhB NPs. **d** Absorption spectra of free RhB and RhB NPs. **e** OPA fluorescence spectra of free RhB and RhB NPs. **f** TPA fluorescence spectra of free RhB with different excitation wavelengths. **g** TPA fluorescence spectra of free RhB and RhB NPs. **h** CLSM image of RhB NPs. The emission range of red fluorescence covered the wavelength from 575 to 630 nm. The scale bar is 10 μm. The RhB concentration used in all figures was 25 μM.

Supplementary Fig. 13

Molecular dynamics simulation of ICG-ZHO binary system.

Supplementary Fig. 14

Absorption spectra of **a** free ICG and **b** ICG NPs at different time intervals. The concentration of ICG was 125 μM.

Supplementary Fig. 15

TEM image of the ICG NPs after incubation in 10% FBS at 37 °C for 24 h. The scale bar is 200 nm.

Supplementary Fig. 16

Absorption spectra of ICG NPs and RhB NPs at different pH conditions.

Supplementary Fig. 17

Viability of MCF-7 cells treated with ICG NPs, free ICG, RhB NPs and free RhB. Error bars denote the standard deviation (n = 6 biologically independent cells). Data are presented as mean values +/- S.D., and *P* values are calculated by one-way ANOVA $^{*}P < 0.05$.

Supplementary Fig. 18

Three-dimensional reconstruction images of tumor sections along Z axis (upper), and focal plane images (lower) in 4T1, CT26 and Hela tumor models. The Z depth is 20

μm. All mice were intravenously injected with ICG NPs (250 μM, 200 μL) and the images were collected at 24 h post-injection. The scale bar is 50 μm.

Supplementary Fig. 19

CLSM images of biopsies of tumor tissue extracted from mice after intravenous injection of ICG NPs (250 μM, 200 μL) at 24 h. **a** Fluorescence field image, **b** bright field image and **c** merged field. The images showed that the ICG NPs were taken up by the tumor cells and evenly distributed in the cellular cytoplasm. The scale bar is 250 μm.

Supplementary Fig. 20

OPA fluorescence images of mice at 24 h after intravenous injection of ICG NPs (250 μM, 200 μL).

Captions for Supplementary Tables

Supplementary Tab. 1

Encapsulation of ICG in NPs at different concentrations with addition of 2.640 mM ZHO.

Supplementary Tab. 2

Size, polydispersity index (PDI) and zeta potential (ζ) of NPs, where the PDI is the key parameter to describe the heterogeneity of sample size.

Supplementary Tab. 3

Slopes of fitted line from free NIR cyanine dyes and NPs, where the line is fitted by double exponential model between the emission intensity log (I) and excitation power log (P).

Supplementary Tab. 4

TPA cross section of free RhB, RhB NPs, free ICG and ICG NPs at 808 nm excited by a Ti: Sapphire oscillator laser. RhB dissolved in methanol was used as a reference.

Supplementary Tab. 5

Size, PDI and ζ of ICG NPs before and after incubation in 10% FBS at 37 ^{o}C for 24 h.

Supplementary Tab. 6

The comparison of ICG NPs to other TPA probes.

Captions for Supplementary Movie

Supplementary Movie 1

Three-dimensional reconstruction of tumor site at 24 h after intravenous injection of ICG NPs.

Supplementary Figures

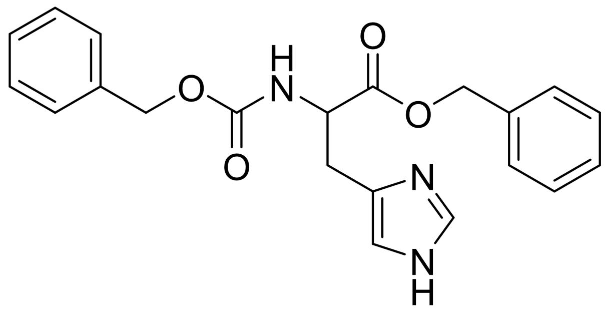

Supplementary Fig. 1 Chemical structure of ZHO.

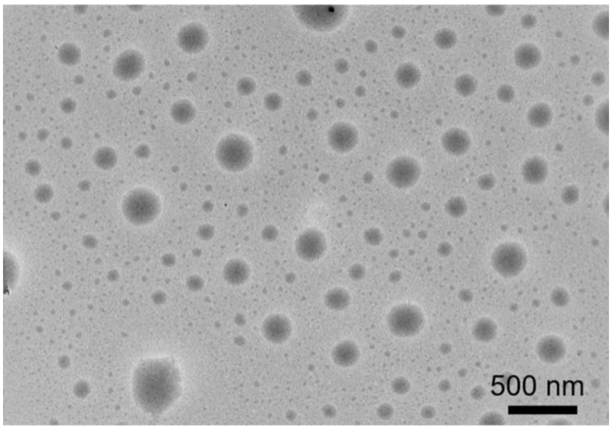

Supplementary Fig. 2 TEM image of ICG NDs.

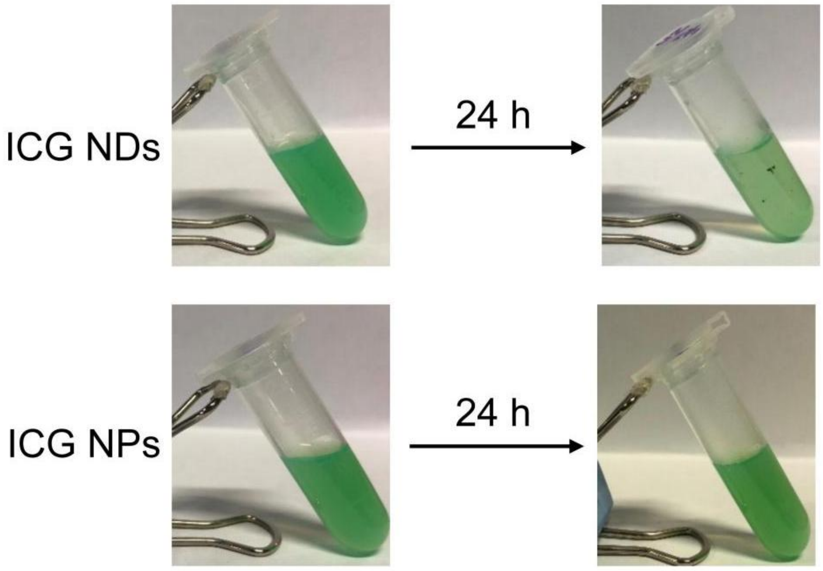

Supplementary Fig. 3 Picture of ICG NDs and ICG NPs before and after aging for 24 h. The concentration of ICG was 125 μM.

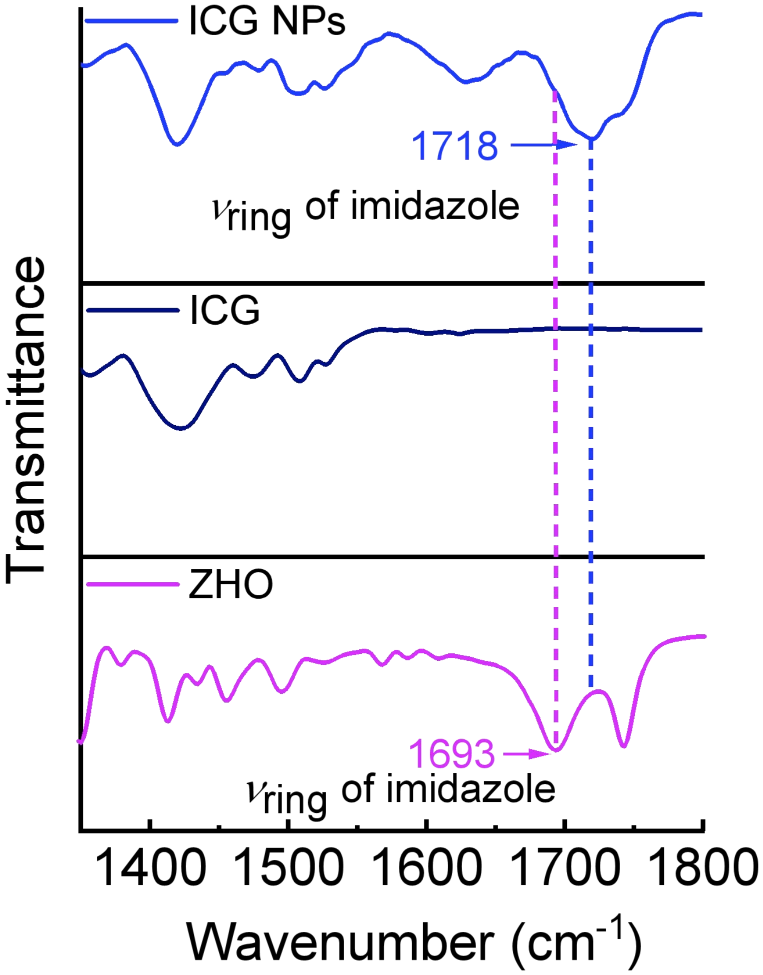

Supplementary Fig. 4 FTIR transmittance spectra of ZHO, ICG and ICG NPs.

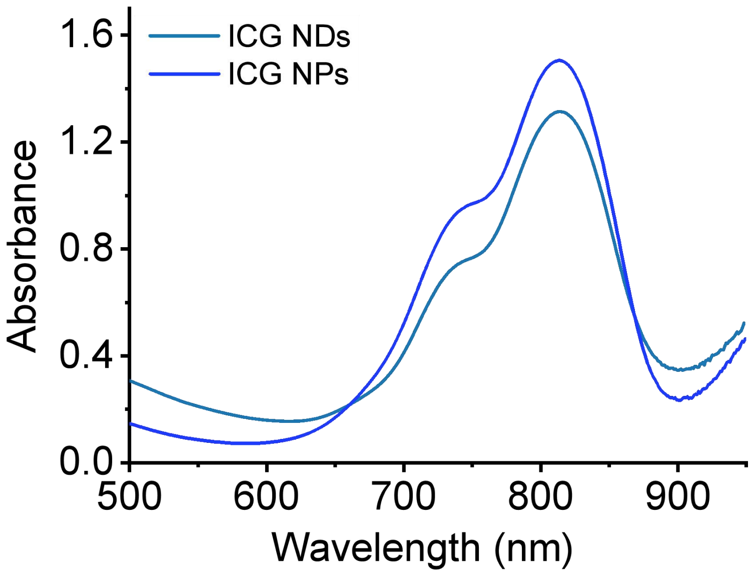

Supplementary Fig. 5 Absorption spectra of ICG NDs and ICG NPs after aging for 24 h. The concentration of ICG was 125 μM.

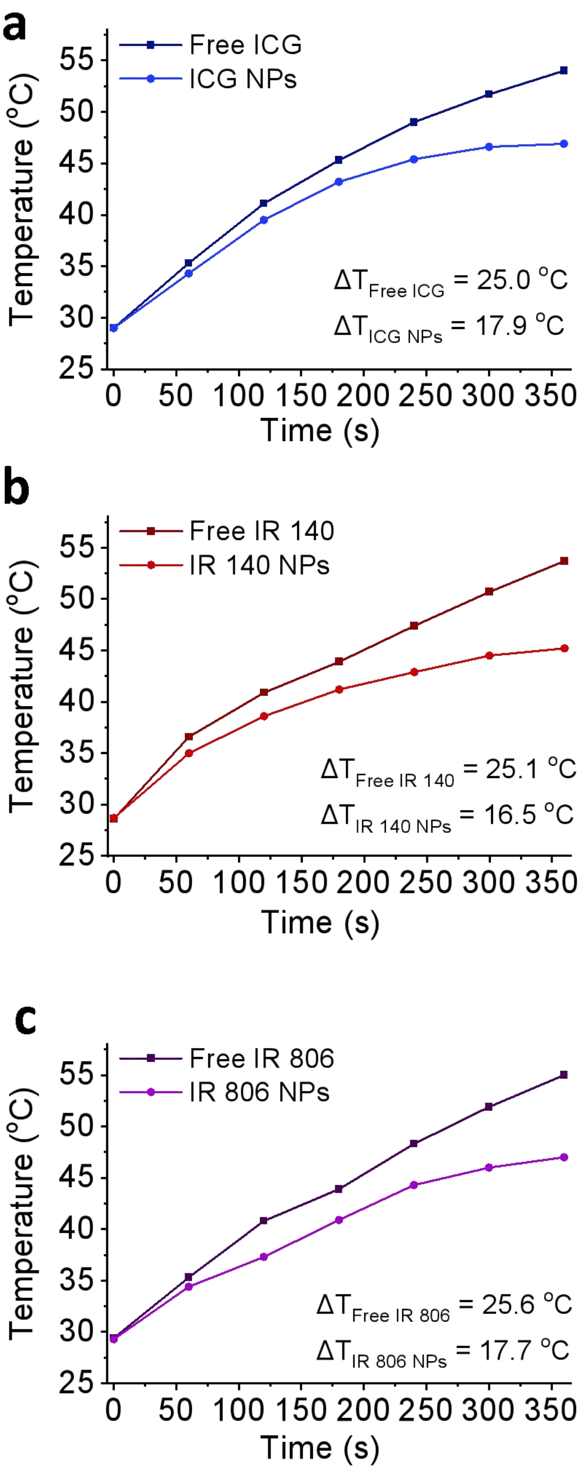

Supplementary Fig. 6 Photothermal relaxation of **a** free ICG and ICG NPs, **b** free IR 140 and IR 140 NPs and **c** free IR 806 and IR 806 NPs. The concentration of NIR cyanine dyes was 25 μM. Samples were excited by 808 nm laser with a power density of 1.5 W cm^{-2}.

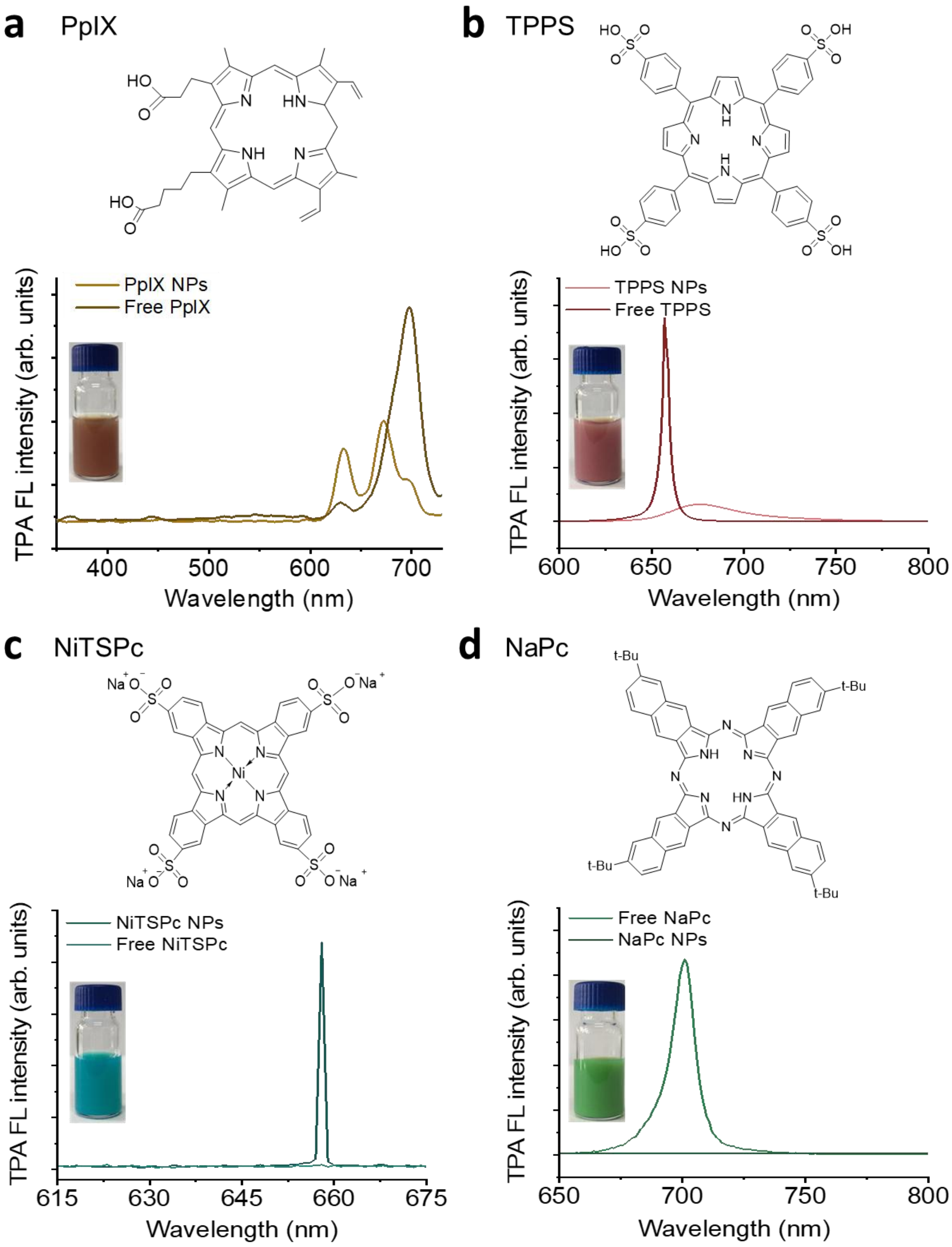

Supplementary Fig. 7 TPA fluorescence spectra of free porphyrin-based dyes (**a** PpIX; **b** TPPS), phthalocyanine-based dyes (**c** NiTSPc; **d** NaPc) and their corresponding NPs, where the PpIX was excited at 780 nm, TPPS was excited at 780 nm, NiTSPc was excited at 780 nm and NaPc was excited at 800 nm. The insert in spectra showed the prepared NPs. The concentration of all dyes used in all figures was 25 μM.

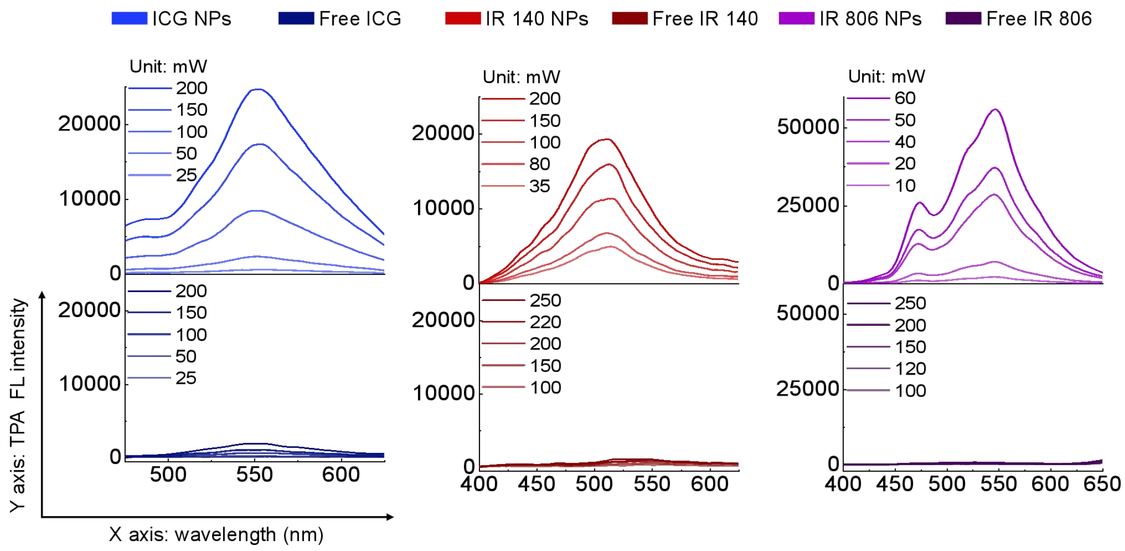

Supplementary Fig. 8 TPA fluorescence spectra of free NIR cyanine dyes and their corresponding NPs as a function of excitation power. The concentration of NIR cyanine dyes and RhB used in all figures was 25 μM.

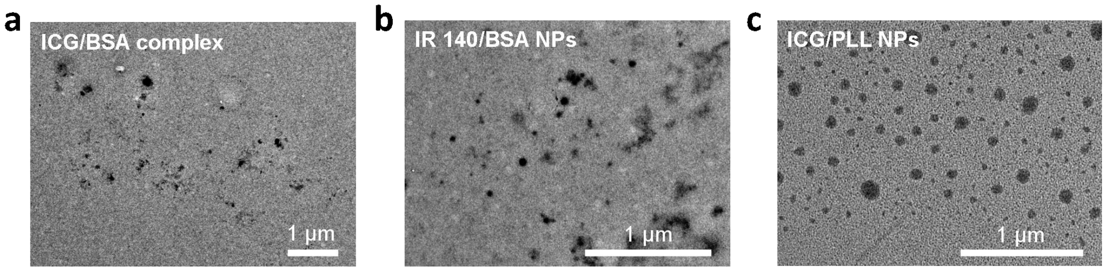

Supplementary Fig. 9 TEM images of **a** ICG/BSA complex, **b** IR 140/BSA NPs and **c** ICG/PLL NPs.

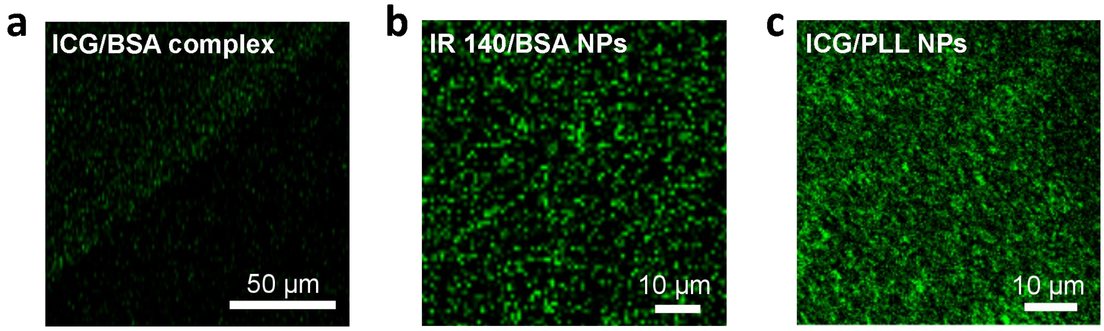

Supplementary Fig. 10 CLSM images of **a** ICG/BSA complex, scale bar is 50 µm, **b** IR 140/BSA NPs, scale bar is 10 µm, and **c** ICG/PLL NPs, scale bar is 10 µm. All figures are obtained in the emission range of 495-540 nm.

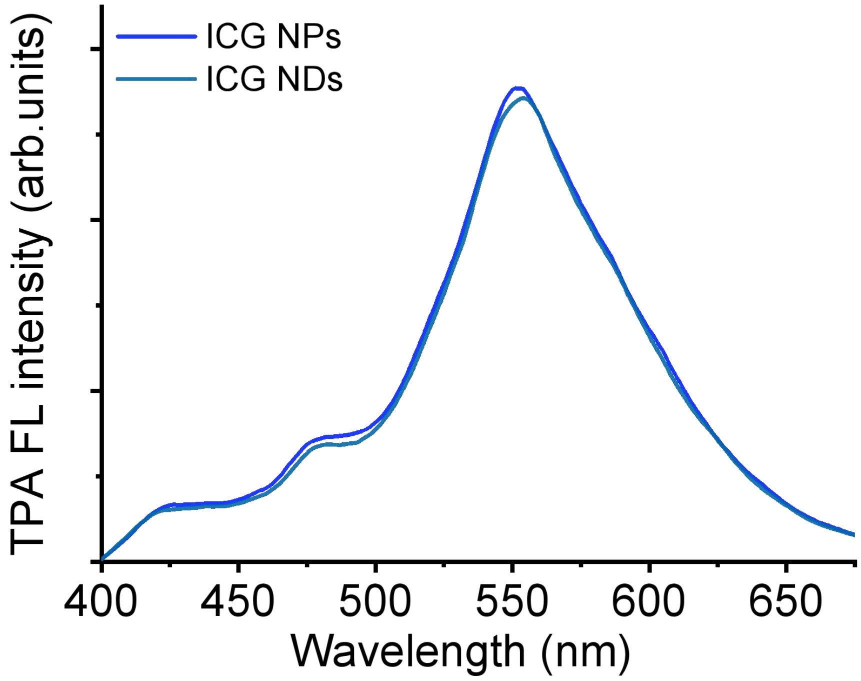

Supplementary Fig. 11 TPA fluorescence spectra of ICG NDs and ICG NPs after aging for 24 h. The concentration of ICG was 25 μM.

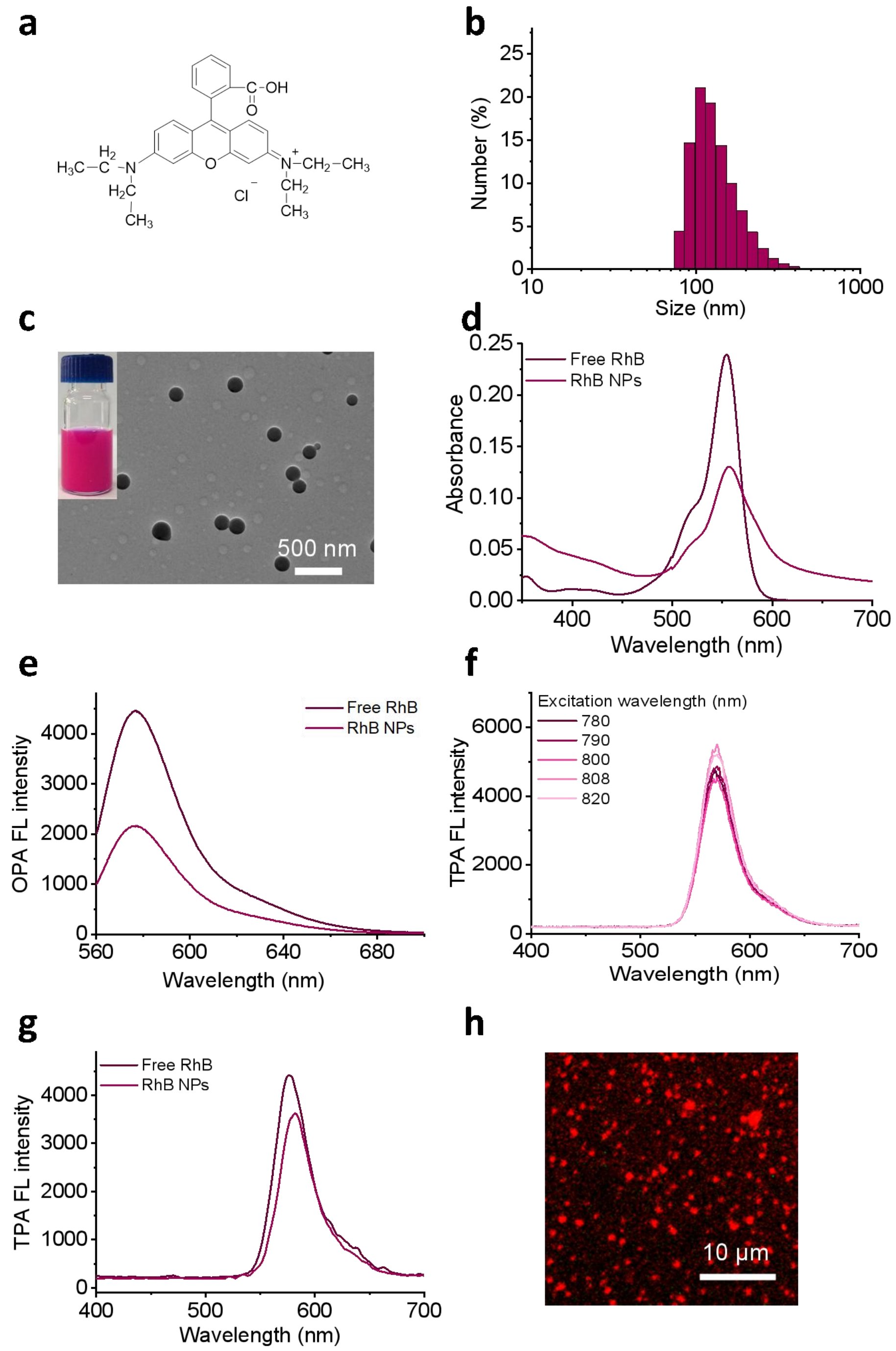

Supplementary Fig. 12 a Chemical structure of RhB. **b** DLS measurements of RhB NPs. **c** TEM image of RhB NPs. The insert shows a picture of the RhB NPs. **d** Absorption spectra of free RhB and RhB NPs. **e** OPA fluorescence spectra of free RhB and RhB NPs. **f** TPA fluorescence spectra of free RhB with different excitation wavelengths. **g** TPA fluorescence spectra of free RhB and RhB NPs. **h** CLSM image of RhB NPs. The emission range of red fluorescence covered the wavelength from 575 to 630 nm. The scale bar is 10 μm. The RhB concentration used in all figures was 25 μM.

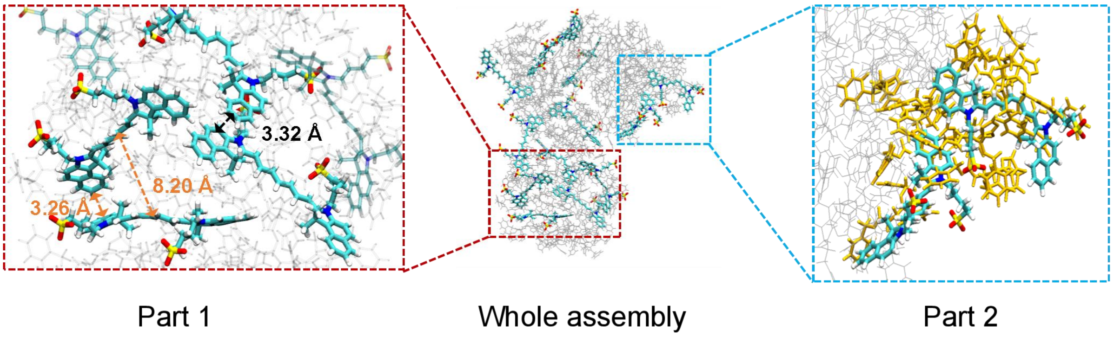

Supplementary Fig. 13 Molecular dynamics simulation of ICG-ZHO binary system. The results revealed that after 50 ns of kinetic simulation, all solute molecules assembled together (whole assembly). In the final assembly, the ICG molecules were found to be distributed on the surface, which can be attributed to the presence of hydrophilic and charged sulfonic acid group in ICG. The ICG molecules exhibited a minimum intermolecular distance of approximately 3.3 Å (Part 1), facilitating electron delocalization for TPA fluorescence emission. Additionally, ICG and ZHO molecules tightly assembled with intermolecular distances less than 3.0 Å (Part 2). Based on energy analysis, we found that the self-assembly of ICG and ZHO is driven by the synergistic effect of hydrophobic effects and electrostatic interactions. Hydrophobic effects significantly contribute to the assembly with an energy of -64.4 kcal mol^{-1} per ICG molecule, which is the major driving force, possibly due to the statistically weak positive charge of ZHO. Comparatively, electrostatic interactions contribute less significantly with an energy of -13.3 kcal mol^{-1} per ICG molecule. No hydrogen bonding interaction was observed in their assembly. ICG molecules were highlighted by blue colors and ZHO molecules were highlighted in yellow.

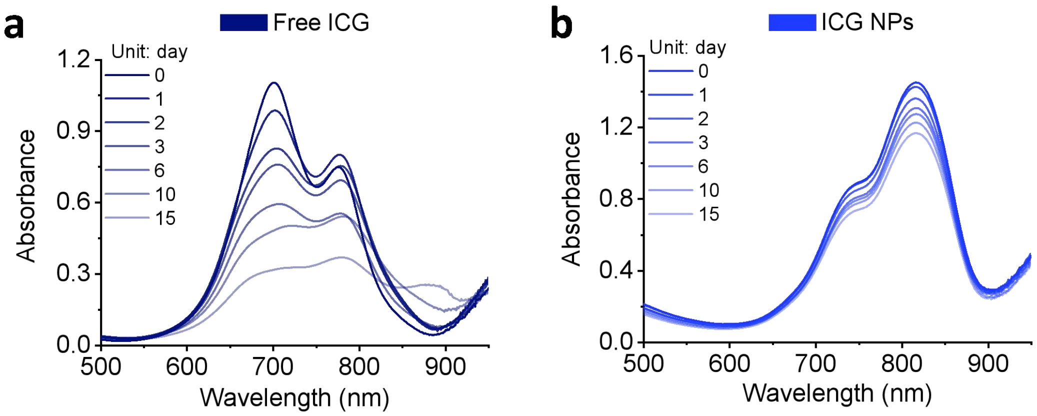

Supplementary Fig. 14 Absorption spectra of **a** free ICG and **b** ICG NPs at different time intervals. The concentration of ICG was 125 μM.

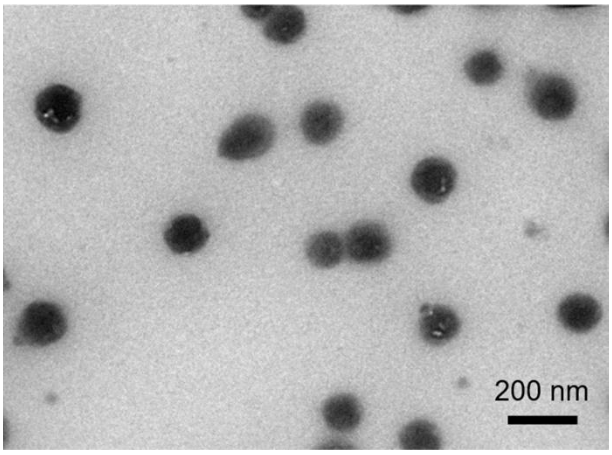

Supplementary Fig. 15 TEM image of the ICG NPs after incubation in 10% FBS at 37 ^{o}C for 24 h. The scale bar is 200 nm.

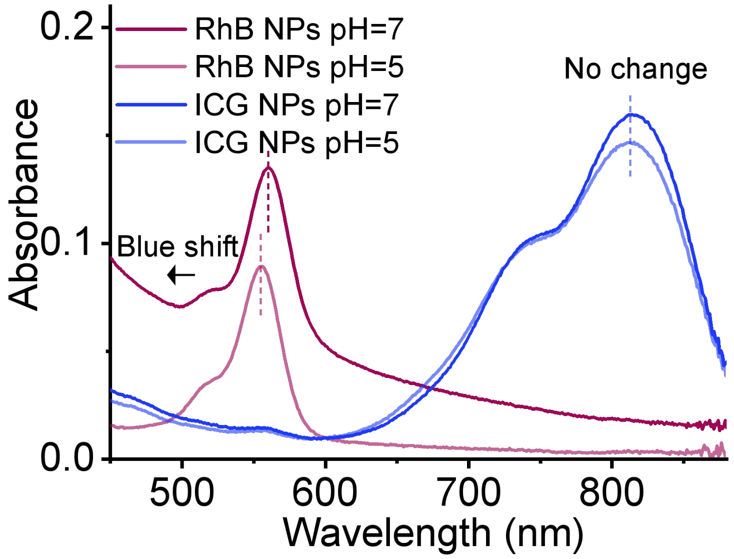

Supplementary Fig. 16 Absorption spectra of ICG NPs and RhB NPs at different pH conditions.

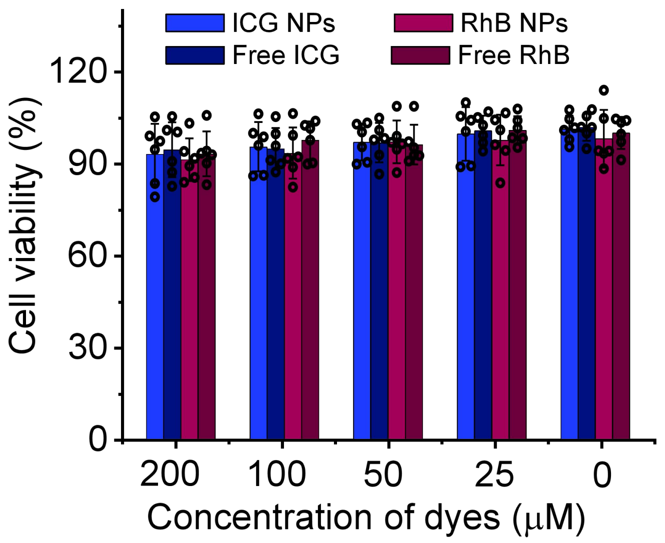

Supplementary Fig. 17 Viability of MCF-7 cells treated with ICG NPs, free ICG, RhB NPs and free RhB. Error bars denote the standard deviation (n = 6 biologically independent cells). Data are presented as mean values +/- S.D., and *P* values are calculated by one-way ANOVA $^{*}P < 0.05$.

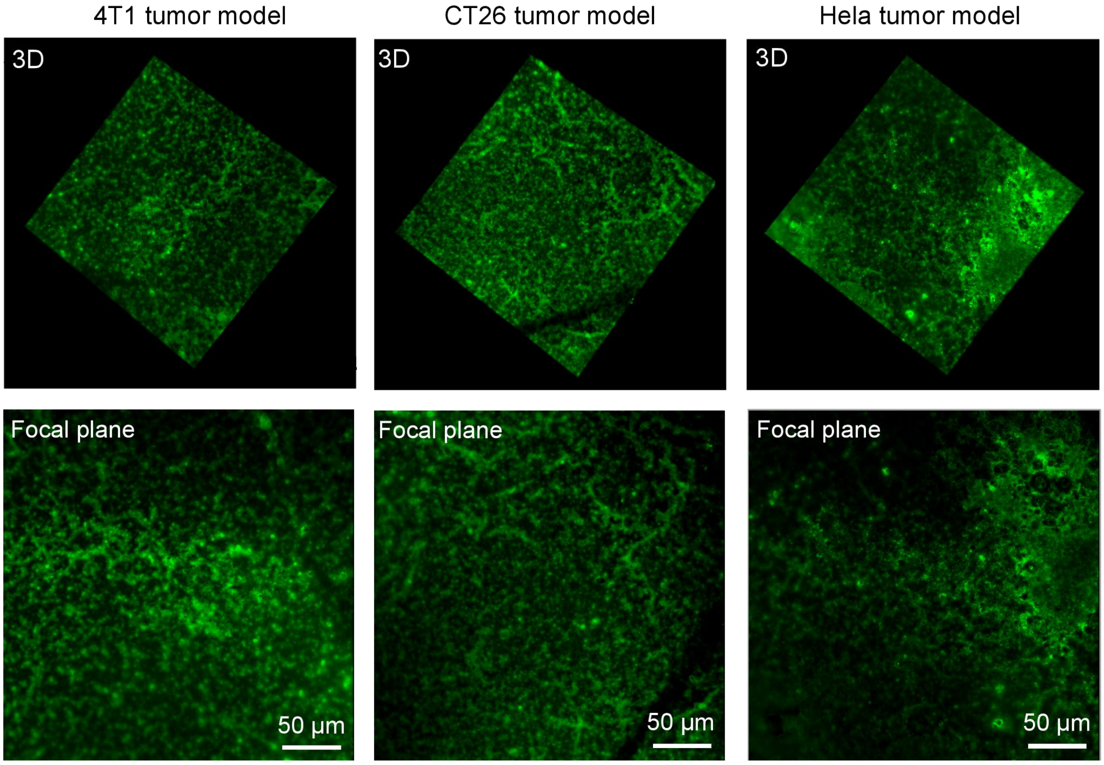

Supplementary Fig. 18 Three-dimensional reconstruction images of tumor sections along Z axis (upper), and focal plane images (lower) in 4T1, CT26 and Hela tumor models. The Z depth is 20 μm. All mice were intravenously injected with ICG NPs (250 μM, 200 μL) and the images were collected at 24 h post-injection. The scale bar is 50 μm.

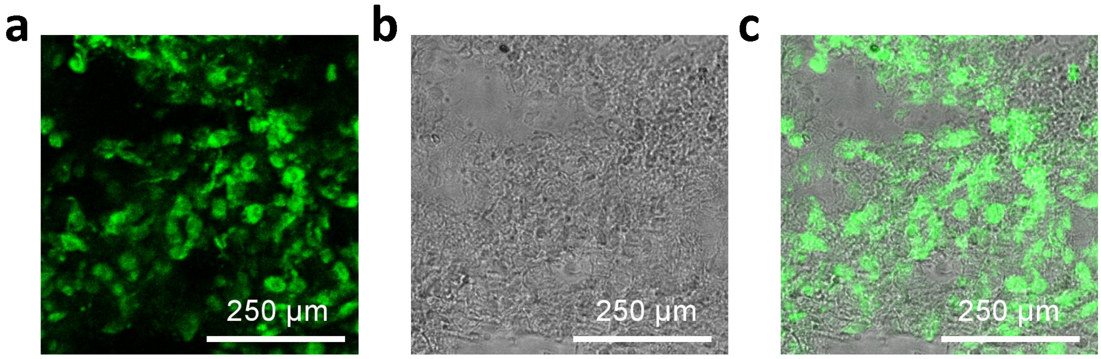

Supplementary Fig. 19 CLSM images of biopsies of tumor tissue extracted from mice after intravenous injection of ICG NPs (250 μM, 200 μL) at 24 h. **a** Fluorescence field image, **b** bright field image and **c** merged field. The images showed that the ICG NPs were taken up by the tumor cells and evenly distributed in the cellular cytoplasm. The scale bar is 250 μm.

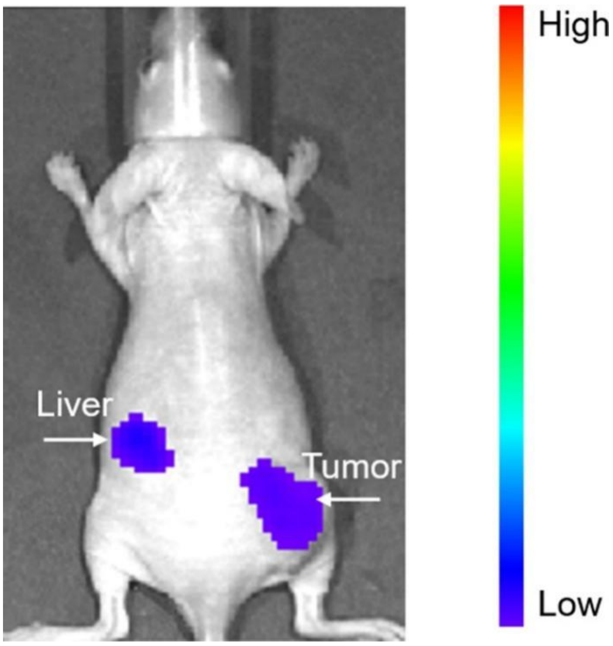

Supplementary Fig. 20 OPA fluorescence images of mice at 24 h after intravenous injection of ICG NPs (250 μM, 200 μL).

Supplementary Tables

Supplementary Tab. 1 Encapsulation of ICG in NPs at different concentrations with addition of 2.640 mM ZHO.

ZHO (mM)		ICG (mM)			
Added	Detected	Added	Detected	EE (%)	LE (%)
2.640	0.870	0.015	0.015	100.0	3.4
	1.203	0.032	0.032	100.0	5.2
	1.232	0.065	0.065	100.0	9.7
	2.020	0.129	0.125	96.9	11.2
	2.381	0.258	0.201	77.9	14.8

Supplementary Tab. 2 Size, polydispersity index (PDI) and zeta potential (ζ) of NPs, where the PDI is the key parameter to describe the heterogeneity of sample size.

NPs	ICG NPs	IR 140	IR 806
Size (nm)	159.8 ± 48.1	132.2 ± 42.1	123.5 ± 56.7
PDI	0.059	0.092	0.274
ζ (mV)	-19.1	-11.4	-12.7

Supplementary Tab. 3 Slopes of fitted line from free NIR cyanine dyes and NPs, where the line is fitted by double exponential model between the emission intensity log (I) and excitation power log (P).

ICG		IR 140		IR 806	
Free	NPs	Free	NPs	Free	NPs
2.0	1.9	1.6	1.5	1.8	1.8

Supplementary Tab. 4 TPA cross section of free RhB, RhB NPs, free ICG and ICG NPs at 808 nm excited by a Ti: Sapphire oscillator laser. RhB dissolved in methanol was used as a reference.

Groups		TPACS (GM)
RhB	Free	121.39
	NPs	99.81
ICG	Free	7.99
	NPs	286.45

Supplementary Tab. 5 Size, PDI and ζ of ICG NPs before and after incubation in 10% FBS at 37 ^{o}C for 24 h.

Incubation	Before	After
Size (nm)	160.5 ± 56.7	163.1 ± 75.9
PDT	0.140	0.244
ζ (mV)	-19.1	-11.8

Supplementary Tab. 6 The comparison of ICG NPs to other TPA probes.

TPA probes	Fabrication		Property		Water solubility or dispersibility	Fluorescence upon aggregation	Fluorescence upon photo-oxidation	Ref. in main text
			Organic	Inorganic				
M-NPs	√	√	√	/	Dispersible	/	/	17
Bis-1,8-naphthalimides based molecular probes	√	/	√	/	Insoluble	Quenched	/	18
Au NPs	/	√	/	√	Dispersible	/	/	19
BDSA/ORMOSIL composite nanoparticles	√	√	√	√	Dispersible	Enhanced	/	20
TPNF	√	√	√	/	Dispersible	/	/	21
BTPETQ dots	√	√	√	/	Dispersible	Enhanced	/	23
2TPAT-AN	√	√	√	/	Dispersible	Enhanced	/	24
C-Dots	/	√	√	√	Dispersible	/	/	26
FeSe QDs	/	√	√	√	Dispersible	/	/	27
Bilayer CrI_3	/	√	/	√	/	/	/	28
pSiNPs	/	√	√	√	Dispersible	/	/	29
ICG NPs	/	√	√	/	Dispersible	Enhanced	Enhanced	This work

/: not applicable or not mentioned

Supplementary Movie

Supplementary Movie 1

Three-dimensional reconstruction of tumor site at 24 h after intravenous injection of ICG NPs.
