# Supplementary material for: Two-photon nanoprobes based on bioorganic nanoarchitectonics with a photo-oxidation enhanced emission mechanism
Source: Nat Commun. 2023 Aug 26;14:5227. doi: 10.1038/s41467-023-40897-4 (PMC10460436; doi:10.1038/s41467-023-40897-4)
Supplement: Supplementary file 2 — Reporting Summary [file 41467_2023_40897_MOESM2_ESM.pdf]

nature portfolio

Corresponding author(s): Xuehai Yan

Last updated by author(s): Aug 3, 2023



# Reporting Summary

Nature Portfolio wishes to improve the reproducibility of the work that we publish. This form provides structure for consistency and transparency in reporting. For further information on Nature Portfolio policies, see our Editorial Policies and the Editorial Policy Checklist.

## Statistics

For all statistical analyses, confirm that the following items are present in the figure legend, table legend, main text, or Methods section.

| n/a | Confirmed | |
|---|---|---|
| ☐ | ☒ | The exact sample size ($n$) for each experimental group/condition, given as a discrete number and unit of measurement |
| ☐ | ☒ | A statement on whether measurements were taken from distinct samples or whether the same sample was measured repeatedly |
| ☐ | ☒ | The statistical test(s) used AND whether they are one- or two-sided<br>*Only common tests should be described solely by name; describe more complex techniques in the Methods section.* |
| ☒ | ☐ | A description of all covariates tested |
| ☐ | ☒ | A description of any assumptions or corrections, such as tests of normality and adjustment for multiple comparisons |
| ☐ | ☒ | A full description of the statistical parameters including central tendency (e.g. means) or other basic estimates (e.g. regression coefficient) AND variation (e.g. standard deviation) or associated estimates of uncertainty (e.g. confidence intervals) |
| ☐ | ☒ | For null hypothesis testing, the test statistic (e.g. $F$, $t$, $r$) with confidence intervals, effect sizes, degrees of freedom and $P$ value noted<br>*Give P values as exact values whenever suitable.* |
| ☒ | ☐ | For Bayesian analysis, information on the choice of priors and Markov chain Monte Carlo settings |
| ☒ | ☐ | For hierarchical and complex designs, identification of the appropriate level for tests and full reporting of outcomes |
| ☒ | ☐ | Estimates of effect sizes (e.g. Cohen's $d$, Pearson's $r$), indicating how they were calculated |

*Our web collection on statistics for biologists contains articles on many of the points above.*

## Software and code

Policy information about availability of computer code

| | |
|---|---|
| Data collection | S-4800 (Hitachi, Japan) with 10 kV accelerating voltage was used for SEM measurements. TEM was performed by a JEM-1011 (JEOL, Japan) at 100 kV with a drop of sample carefully applied to a carbon-coated copper grid and dried in vacuum. The size distribution and zeta potential were determined using a Zetasizer Nano (Malvern, England). CLSM images were acquired by an FV500 confocal laser scanning microscope (Olympus, Japan) equipped with a Ti: Sapphire oscillator laser (Mai Tai, USA). TPA NPs were excited by the adjustable Ti: Sapphire oscillator laser and the signal channels used were 495-540 nm and 575-630 nm. The absorption spectra were recorded using a UV-2600 spectrophotometer (Shimadzu, Japan) with a quartz cuvette of 1 mm path length. The F-4500 fluorescence spectrometer (Hitachi, Japan) equipped with Xenon lamp as excitation source was used to measure the OPA fluorescence spectra of the samples with a quartz cuvette of 1.0 cm. The TPA fluorescence spectra were measured on a home-made optical platform. For excitation, an adjustable fs Ti: Sapphire oscillator laser (100 fs, SP-5W, Spectra physics, America) equipped with a short-pass filter (730 nm) was applied onto the sample in a 1.0 cm quartz cuvette, and spectra were recorded by an Omni-λ300 monochromator/spectrograph (Zolix, China) equipped with a PMTH-S1C1-CR131 photomultiplier tube. EPR measurements were conducted on the ESP-300 spectrometer (Bruker, America), which equipped with 808 nm laser at room temperature. TEMP agent was added into the samples to capture the singlet oxygen signal. FTIR spectra were recorded by the TENSOR 27 FTIR spectrometer (Bruker, America), with the samples prepared using the KBr pellet method. The molecular weight mass charge ratio (m/z) of ICG NPs were determined by the SolariX ESI-MS (Bruker, America). For photothermal relaxation test, the temperature increase was recorded every 60 s with the UT320 digital thermometer (UNI-T, China), and samples were irradiated by 808 nm laser with a power density of 1.5 W cm-2. |
| Data analysis | Statistical analysis were carried out via Origin 2022. |

For manuscripts utilizing custom algorithms or software that are central to the research but not yet described in published literature, software must be made available to editors and reviewers. We strongly encourage code deposition in a community repository (e.g. GitHub). See the Nature Portfolio guidelines for submitting code & software for further information.

## Data

Policy information about availability of data

All manuscripts must include a data availability statement. This statement should provide the following information, where applicable:

- Accession codes, unique identifiers, or web links for publicly available datasets
- A description of any restrictions on data availability
- For clinical datasets or third party data, please ensure that the statement adheres to our policy

The Source data for main and supplementary figures generated in this study have been deposited in Figshare (https://figshare.com/s/15318812234a7e3721ba). The full image dataset is available from the corresponding author upon request. The remaining data are available within the Article, Supplementary Information or Source data file. Source data are provided with this paper.

## Human research participants

Policy information about studies involving human research participants and Sex and Gender in Research.

| | |
|---|---|
| Reporting on sex and gender | Human research participants are not involved, therefore the reporting on sex and gender is not applicable in the study. |
| Population characteristics | Human research participants are not involved, therefore the population characteristics are not applicable in the study. |
| Recruitment | No human research participants are recruited in the study. |
| Ethics oversight | Ethics oversight of human research participants is not applicable in the study. |

Note that full information on the approval of the study protocol must also be provided in the manuscript.

# Field-specific reporting

Please select the one below that is the best fit for your research. If you are not sure, read the appropriate sections before making your selection.

☒ Life sciences ☐ Behavioural & social sciences ☐ Ecological, evolutionary & environmental sciences

For a reference copy of the document with all sections, see nature.com/documents/nr-reporting-summary-flat.pdf

# Life sciences study design

All studies must disclose on these points even when the disclosure is negative.

| | |
|---|---|
| Sample size | In this study, sample size was determined by referring to pilot studies and relevant literature. For a standard MTT assay, we used 6 biological replicates per group. For the experiments including in vitro cell imaging, in vivo mice imaging and tumor histological analysis, the sample size was 3 independently biological replicates each group. |
| Data exclusions | No data was excluded from the analyses. |
| Replication | Experiments were replicated 3 times and obtained the similar results. |
| Randomization | Samples and tumor-bearing mice were randomly allocated into experimental groups. |
| Blinding | The investigators were blinded to group allocation during data collection and/or analysis. |

# Reporting for specific materials, systems and methods

We require information from authors about some types of materials, experimental systems and methods used in many studies. Here, indicate whether each material, system or method listed is relevant to your study. If you are not sure if a list item applies to your research, read the appropriate section before selecting a response.

## Materials & experimental systems

| n/a | Involved in the study |
| --- | --- |
| ☒ | ☐ Antibodies |
| ☐ | ☒ Eukaryotic cell lines |
| ☒ | ☐ Palaeontology and archaeology |
| ☐ | ☒ Animals and other organisms |
| ☒ | ☐ Clinical data |
| ☒ | ☐ Dual use research of concern |

## Methods

| n/a | Involved in the study |
| --- | --- |
| ☒ | ☐ ChIP-seq |
| ☒ | ☐ Flow cytometry |
| ☒ | ☐ MRI-based neuroimaging |

# Eukaryotic cell lines

Policy information about cell lines and Sex and Gender in Research

| | |
| --- | --- |
| Cell line source(s) | MCF-7 cells, CT 26 cells, 4T1 cells and Hela cells used in the study were provided by the National Collection of Authenticated Cell Cultures. |
| Authentication | Cells lines used in the study are commercial available, and they are authenticated by STR profiling. |
| Mycoplasma contamination | MCF-7 cell line, CT 26 cell line, 4T1 cell line and Hela cell line used in the study were tested negative for mycoplasma contamination. |
| Commonly misidentified lines (See ICLAC register) | No commonly misidentified cell lines were used in the study. |

# Animals and other research organisms

Policy information about studies involving animals; ARRIVE guidelines recommended for reporting animal research, and Sex and Gender in Research

| | |
| --- | --- |
| Laboratory animals | Female BALB/c nude mice with age of 6-8 weeks old were provided by Beijing HFK Bioscience Co. Ltd. in the study. The mice were housed in an environmentally controlled animal facility (temperature 23 °C, humidity 55 ± 5 %) with regular 12/12 cycle. |
| Wild animals | The study did not involve wild animals. |
| Reporting on sex | The sex of mice used in the study was female. |
| Field-collected samples | The study did not involve field-collected samples. |
| Ethics oversight | All animal experiments were conducted in accordance to the protocols approved by the Ethics Committee of the Institute of process engineering, Chinese Academy of Sciences (permit number: IPEAECA2018061). |

Note that full information on the approval of the study protocol must also be provided in the manuscript.
