# Supplementary material for: Two-photon nanoprobes based on bioorganic nanoarchitectonics with a photo-oxidation enhanced emission mechanism
Source: Nat Commun. 2023 Aug 26;14:5227. doi: 10.1038/s41467-023-40897-4 (PMC10460436; doi:10.1038/s41467-023-40897-4)
Supplement: Supplementary file 3 — Description of Additional Supplementary Files [file 41467_2023_40897_MOESM3_ESM.pdf]

Title: Supplementary Movie 1
Description: Threedimensional reconstruction of tumor site at 24 h after intravenous injection of ICG NPs.
